# Lanthanides Singing the Blues: Their Fascinating Role in the Assembly of Gigantic Molybdenum Blue Wheels

**DOI:** 10.1021/acsnanoscienceau.1c00036

**Published:** 2022-03-02

**Authors:** Emir Al-Sayed, Annette Rompel

**Affiliations:** Universität Wien, Fakultät für Chemie, Institut für Biophysikalische Chemie, Althanstraße 14, 1090 Wien, Austria

**Keywords:** polyoxomolybdate, hybrid organic−inorganic, stereoselectivity, catalysis, molecular recognition, lanthanide separation

## Abstract

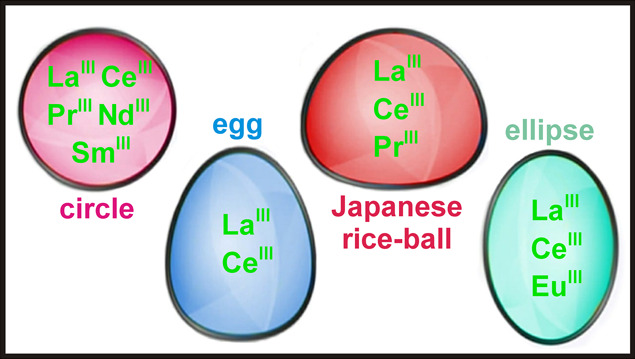

Molybdenum blues (MBs) are a distinct class of polyoxometalates, exhibiting versatile/impressive architectures and high structural flexibility. In acidified and reduced aqueous environments, isopolymolybdates generate precisely organizable building blocks, which enable unique nanoscopic molecular systems (MBs) to be constructed and further fine-tuned by hetero elements such as lanthanide (Ln) ions. This Review discusses wheel-shaped MB-based structure types with strong emphasis on the ∼30 Ln-containing MBs as of August 2021, which include both organically hybridized and nonhybridized structures synthesized to date. The spotlight is thereby put on the lanthanide ions and ligand types, which are crucial for the resulting Ln-patterns and alterations in the gigantic structures. Several critical steps and reaction conditions in their synthesis are highlighted, as well as appropriate methods to investigate them both in solid state and in solution. The final section addresses the homogeneous/heterogeneous catalytic, molecular recognition and separation properties of wheel-shaped Ln-MBs, emphasizing their inimitable behavior and encouraging their application in these areas.

## 1 Introduction

**Figure 1:**
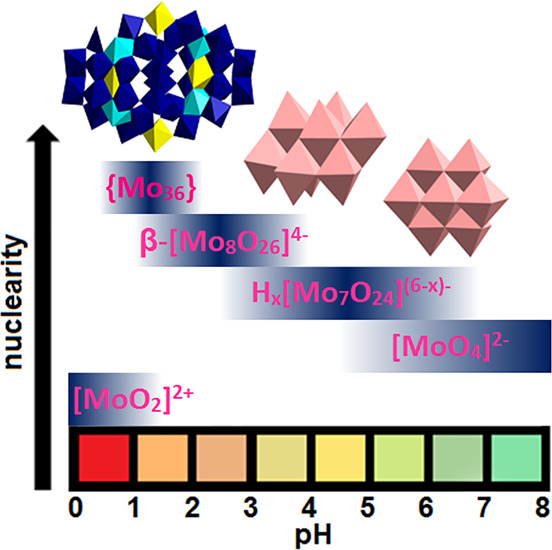
IPOMos speciation in an aqueous solution with a Mo^VI^ concentration of 0.1–0.4 M.^4^ The maximum strength of the blue color in each bar reflects the maximum concentration of the corresponding species in the respective pH region. The arrangement of the blue bars is merely based on the increasing nuclearity of the molybdate clusters and does not display any dominance over other species at a given pH range. *x* in H_*x*_[Mo_7_O_24_]^(6–*x*)–^ varies between 0 and 2. The crystallized IPOMos [Mo_7_O_24_]^6–^, β-[Mo_8_O_26_]^4–^, and [Mo_36_O_112_(OH_2_)_16_]^8–^ ({Mo_36_}) are depicted in polyhedral representation. Coloring code: {MoO_6_}, rose and yellow; {Mo_8_O_35_}, blue with central {MoO_7_}-unit in cyan.

Orthomolybdate ([MoO_4_]^2–^) solutions exhibit a unique chemistry as they allow for the assembly of nanosized scaffolds with the general formula [X_*a*_Y_*b*_H_*c*_Mo^VI^_*x*_Mo^V^_*y*_O_*z*_(H_2_O)_*v*_]^*n*−^ ({Mo_*w*_X_*a*_Y_*b*_} (*a* = number of organic ligands; *b* = number of metallic hetero elements; *c* = degree of protonation; *x* and *y* = number of unreduced and reduced molybdenum, respectively; *z* = number of oxygens; *v* = number of coordinated water; *n* = resulting charge of the nanosized scaffold; *w* = sum of unreduced and reduced molybdenum). They are up to ∼4 nm large (for wheel-type clusters)^1^ and exhibit unprecedented topology.^2^ In a one-pot reaction an aqueous orthomolybdate solution is acidified (pH ≤ 4.5)^3^ to initiate the polymerization process resulting in a number of isopolyoxomolybdates (IPOMos) (Figure 1). Generally, polymerization processes of orthomolybdate [MoO_4_]^2–^ occur in solutions with concentrations greater than 10^–4^ M (Figure 1).^4^ The speciation of [MoO_4_]^2–^ in acidified solutions with different concentrations and ionic strengths has been comprehensively investigated.^5−8^ In strongly alkaline environments, the monomeric [MoO_4_]^2–^ anion predominates, while in strongly acidic solutions the dominant species are stable mono-[MoO_2_(OH)(H_2_O)_3_]^+^ or bi-[Mo_2_O_5_(OH)(H_2_O)_5_]^+^ oxocations able to form inorganic–organic complexes with organic ligands (e.g., dicarboxylic acids, hydroxyaldehydes, amines, etc.).^7,9−14^ The major species formed first upon acidification of aqueous [MoO_4_]^2–^ (Figure 1) is heptamolybdate, H_*x*_[Mo_7_O_24_]^(6–*x*)–^ (Figure 1), which reaches typically its maximum concentration at pH 5 and can be protonated upon further acidification.^4^ When the environment becomes more acidic, the generation of β-[Mo_8_O_26_]^4–^ prevails over H_*x*_[Mo_7_O_24_]^(6–*x*)–^ (*x* = 0–2; max. conc. of H_*x*_[Mo_7_O_24_]^(6–*x*)–^ is reached at pH 4.5) and reaches a maximum at pH ∼ 2.5. [Mo_36_O_112_(OH_2_)_16_]^8–^ {Mo_36_} (Figure 1), which is a dimer of [Mo_18_O_56_(H_2_O)_8_]^4–^ with an inversion center, is the largest known IPOMo present in solution under nonreducing conditions below pH 2.8.^15^

**Figure 2:**
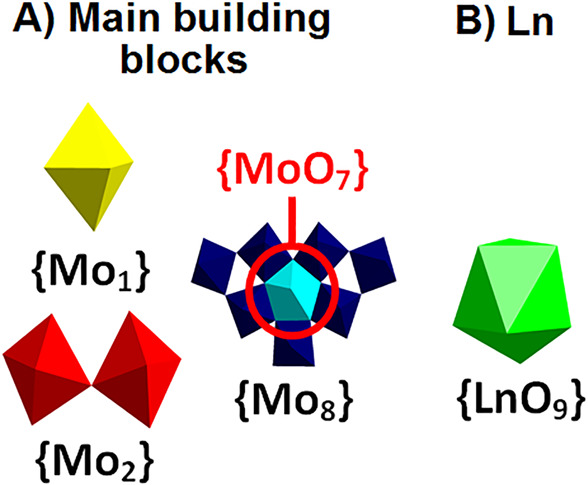
Polyhedral representation of the building blocks and the lanthanide units. (A) Virtual linker units {MoO_6_} ({Mo_1_}) and {Mo_2_O_11_} ({Mo_2_}) and the fundamental building block {Mo_8_O_35_} ({Mo_8_}) form MB rings of different nuclearity.^2^ (B) Lanthanide ions with a tricapped trigonal prismatic coordination sphere. Coloring code: {MoO_6_}, yellow; {Mo_2_O_11_}, red; {Mo_8_O_35_}, blue with central {MoO_7_}-unit in cyan; {LnO_9_}, green.

**Figure 3:**
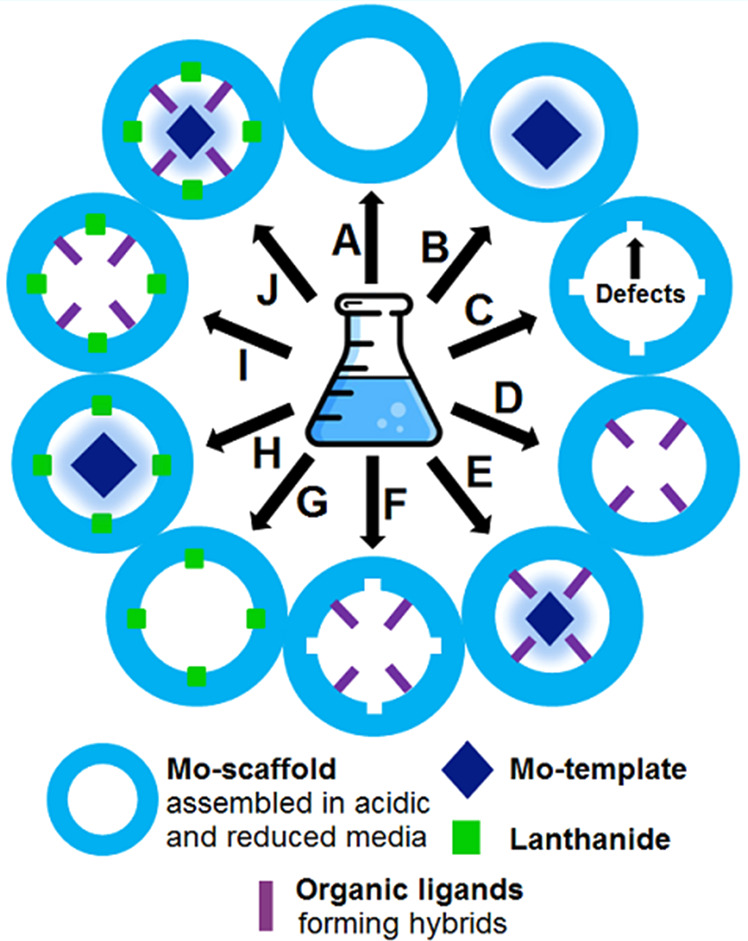
Simplified representation of all available types of MB/Ln-MB-based nanoclusters: (A) Complete MB (described in section 3.1), (B) templated complete MB (section 3.1), (C) lacunary MB (section 3.1), (D) hybridized complete MB (section 3.1), (E) templated and hybridized complete MB (section 3.1), (F) lacunary hybridized MB (section 3.1), (G) complete Ln-MB (sections 3.2.1.1. and 3.2.2.1), (H) templated complete Ln-MB (sections 3.2.1.2. and 3.2.2.2), (I) hybridized complete Ln-MB (sections 3.2.3.1. and 3.2.4.1.), and (J) templated and hybridized complete Ln-MB ring system (sections 3.2.3.2. and 3.2.4.2.). All cluster types are mainly synthesized in a one-pot synthesis, whereby the cluster type (G) may also be produced via a two-step synthesis by first synthesizing the cluster type C and then adding the lanthanide ions.

Upon acidification and subsequent addition of a reducing agent (e.g., N_2_H_4_, Na_2_S_2_O_4_) the orthomolybdate ([MoO_4_]^2–^) solution turns blue and the virtual linker units {MoO_6_} ({Mo_1_}), {Mo_2_O_11_} ({Mo_2_}) (Figure 2A)^16^ and the fundamental building block {Mo_8_O_35_} ({Mo_8_}) with the pentagonal unit {MoO_7_Mo_5_O_20_} ({Mo(Mo)_5_}) (Figure 2A) are formed.^2^ {Mo_1_}, {Mo_2_}, and {Mo_8_} can be linked together to form complex and large wheel-type structures (Figure 3).^2^

Solutions containing gigantic wheel-type clusters based on molybdenum oxide are referred to as “Molybdenum Blues” (MBs).^17^ The reductively triggered blue color is a result of delocalized 4d electrons over the equator of these wheels.^17^ MBs were first reported by Scheele,^18^ but the structure of “amorphous molybdenum blues” remained unknown until the late 20th century when the giant wheel {Mo_154_} (Figure 5A) constructed from the building blocks {Mo_1_}, {Mo_2_}, and {Mo_8_} (Figure 2A) was synthesized and structurally elucidated by Müller et al.^19^ The general formula for discrete pure molybdenum oxide-based wheels is [{Mo^VI^_2_O_5_(H_2_O)_2_}^2+^_*b*–*x*_{(Mo^VI/V^_8_O_26_(μ_3_-O)_2_H_*m*_(H_2_O)_3_Mo^VI/V^)^(4–*m*)–^}_*b*_]^(2*b*−*bm*+2*x*)–^ (*b* = number of building units per set; *m* = number of protons; *x* = number of defect sites).^17^ The pentagonal unit {(Mo)Mo_5_} is responsible for the distinctive circular structure of {Mo_154_}, which comprises a central pentagonal bipyramidal {MoO_7_}-unit (Figure 2A). The crystallization of {Mo_154_} (Figure 5A) was only made possible by destroying its hydration shell with high electrolyte concentrations.^17^

Since 1995, a plethora of nanosized, molecular wheel-shaped clusters has been synthesized (Figure 3), exhibiting different degrees of nuclearity (starting from {Mo_138_}^20^ (Figure 5C) to {Mo_176_}^1^ (Figure 5B)), whereby clusters of lower nuclearity ({Mo_138_} to {Mo_152_})^3^ contain defect sites^3^ (= vacancies within the MBs due to the lack of {Mo_2_}-units) (Figure 5C). {Mo_176_}^1^ is the largest discrete wheel to date, which is constructed of two {Mo_1_}, {Mo_2_}, and {Mo_8_} building blocks more than {Mo_154_}.^19^ The incorporation of these additional building blocks also results in the largest known nanocavity ({Mo_154_} exhibits a 20 Å and {Mo_176_} a 25 Å nanocavity; see Figure 5A and B).

Smaller fragments of suitable size, such as the IPOMo [Mo_36_O_112_(OH_2_)_16_]^8–^ ({Mo_36_}),^15^ can be templated into these wheel-shaped structures by stabilization in the central nanocavity of the wheel via supramolecular interactions, resulting in host–guest systems (e.g., {Mo_36_}⊂{Mo_150_} with the symbol “⊂” representing the inclusion of a template cluster in a ring) (Figure 3B).^21^ This template effect was first realized by the incorporation of two nonisolable {Mo_36_O_96_(H_2_O)_24_} “hubcaps” into the nanocavity of the {Mo_176_}^1^ nanowheel, thereby forming the “nanocookie” {Mo_248_}.^22^

Aside from generating defects in the cluster or introducing templates, another structural modification is the coordinative fixation of organic ligands onto {Mo_2_} ({Mo^VI^_2_O_5_(H_2_O)_2_}^2+^)^17^ units or replacement of {Mo_2_} units by metallic hetero elements of adequate size, such as lanthanide ions (Figure 2B) in the inner rings (the terms inner and outer ring/rim refer to the inner and outer regions of the wheels; Figure 5). The coordinative attachment of organic ligands on MBs was achieved initially by hybridizing {Mo_154_} with cysteines.^23^ This type of MBs (= organic–inorganic hybrids) (Figure 3D) represents a fusion of inorganic, nanosized giant clusters with organic components and possess characteristics that vary from their parent cluster, such as decreased solubility in comparison to the parent wheel^20,24^ and a functionalized nanocavity (Figure 6B).

The integration of lanthanide ions into MBs was accomplished first by the introduction of Pr^III^ into an MB scaffold, forming the wheel {Mo_120_Pr_6_}^25^ (Figure 5D), which exhibited a “Japanese rice-ball”-shape with an outer diameter of ∼31 Å, rather than a circular-shape as the parent wheel {Mo_154_} (Figure 5A).

The incorporation of lanthanide ions in MBs is particularly intriguing since new unique MB characteristics may arise. For example, when complexed, lanthanides can behave like typical Lewis acids and exhibit interesting catalytic properties.^26^ In several organic transformation reactions, lanthanide-containing POMs show significant chemoselectivity.^27,28^ Furthermore, lanthanide ions in POM-based nanostructures are also able to form inorganic–organic adducts, which can be exploited in chiral recognition.^29^

This Review addresses (i) the synthesis and reaction conditions of all available structural types of MB-based wheels (Figure 3) with strong emphasis on their derivatization with lanthanide ions as the fascinating role of lanthanide ions in wheel-type MBs has not been covered yet in a review, (ii) thoroughly the geometrical changes and charge shifts to the parent {Mo_154_} cluster caused by the introduction of lanthanide ions, (iii) the inclusion of templates, and (iv) the introduction and role of (structure directing and chiral-inducing) organic ligands in MB-based architectures. As the main focus is on Ln-MB structures, techniques for confirming their intactness in solution as well as investigating their solution speciation (e.g., “blackberries”^30,31^) are presented using selected examples. The final section is devoted to potential applications of wheel-shaped Ln-MB architectures as homo- and heterogeneous catalysts as well as attractive components for molecular recognition. Additionally, specific Ln-MB syntheses are described as an elegant approach in lanthanide separation.^31^

## 2 General Synthesis Approach of MB/Ln-MB-Based Nanoclusters

The general approach to synthesize all types of MBs/Ln-MBs, which are shown in Figure 3, is quite straightforward. The partial reduction of molybdate(VI) solutions in an acidic environment is shared by all cluster type syntheses. Adjusted molybdate concentrations allow for the production of desirable clusters while preventing the formation of undesired byproducts.^22,32^ A synthetic approach to force these cluster to crystallize is to destroy their hydration shell by a high electrolyte concentration.^17^

The amount of reducing agent has less of an effect on MB formation as the pH of the reaction solution decreases.^33^ Above a pH value of ∼1.5, excessively high amounts of reducing agents (e.g., Na_2_S_2_O_4_) lead to brown reaction mixtures (a compound class containing the structural building block unit O–Mo^V^–O–Mo^V^–O)^17,19,33−36^ rather than blue solutions. Typically, the pH is indirectly proportional to the nuclearity of the cluster (i.e., a decrease of the pH results in an increase of the clusters’ nuclearity)^3^ and the molar ratio of molybdate/additional framework-building reagents (metallic heteroions, ligands) must be optimized for successful crystallization.^32,37^

Organic ligands for the synthesis of hybridized inorganic–organic MBs/Ln-MBs should have a carboxyl group in order to be able to attach to {Mo_2_} units (always depicted as red polyhedra in all the figures). Furthermore, amino groups in ligands (e.g., amino acids and peptides) are of essential importance particularly in Ln-MB syntheses when it comes to (a) stabilization of templates inside the rings,^38^ (b) introduction of chirality,^24^ and (c) modulation of the shape and size of Ln-MBs.^39^

## 3 Synthesis of MB/Ln-MB Based Nanoclusters

### 3.1 MB Based Nanoclusters

**Figure 4:**
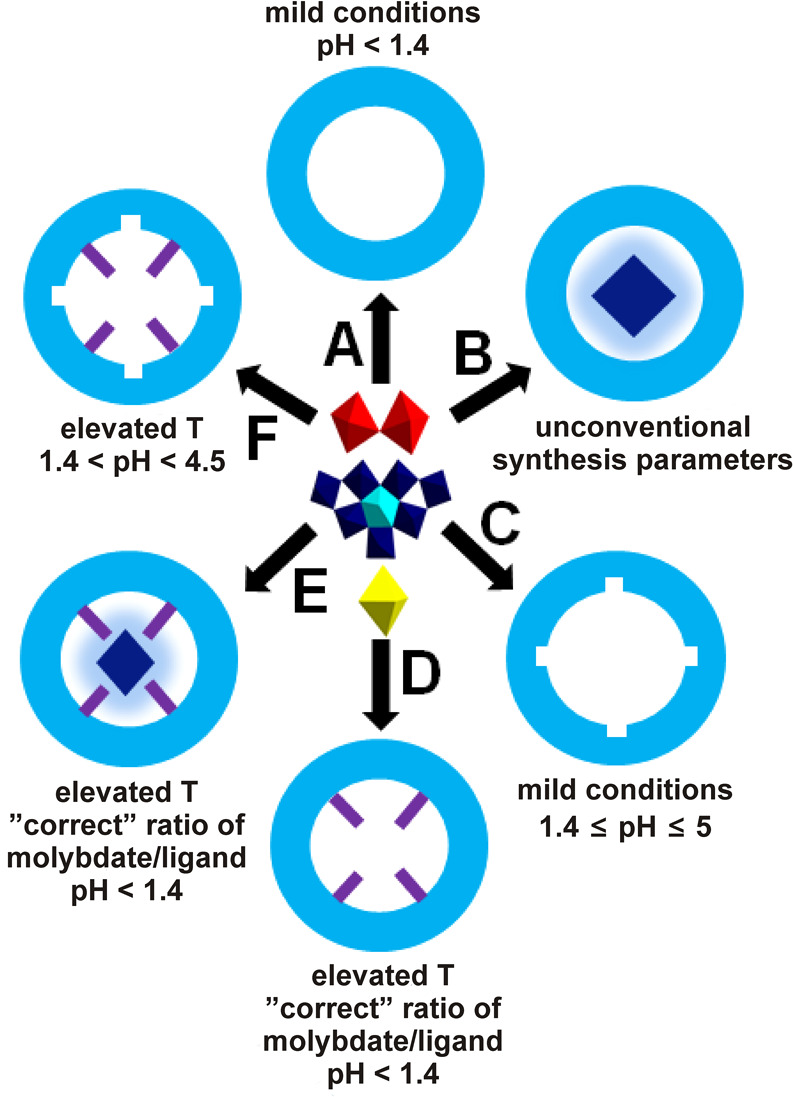
Overview of major synthesis aspects in the production of a (A) complete MB, (B) templated complete MB, (C) lacunary MB, (D) hybridized complete MB, (E) templated and hybridized complete MB ring system, and (F) lacunary hybridized MB ring system (synthesis temperature:T). Coloring code: {MoO_6_}, yellow; {Mo_2_O_11_}, red; {Mo_8_O_35_}, blue with central {MoO_7_}-unit in cyan.

**Figure 5:**
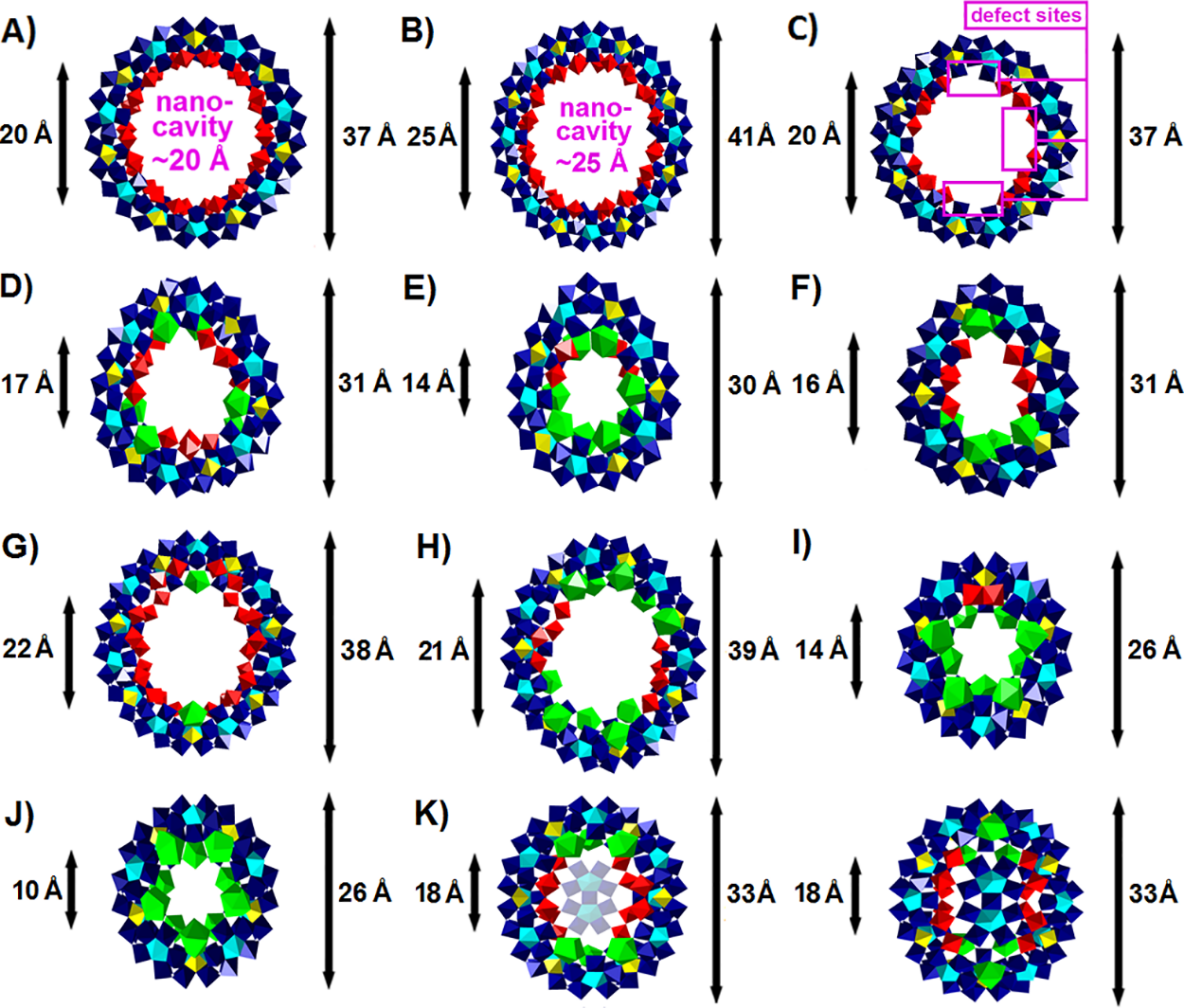
Polyhedral representation (and inner and outer diameter of the respective ring/rim) of the purely inorganic complete MB and all purely inorganic complete Ln-MB ring systems as of August 2021 described in this review starting with the (A) circle-shaped {Mo_154_}^19^ (simplified archetype is shown in Figure 3A), (B) circle-shaped {Mo_176_}^1^ (Figure 3A), (C) circle-shaped {Mo_138_}^20^ (Figure 3C), (D) “Japanese rice-ball” {Mo_120_Ln_6_}^40^ (Figure 3G), (E) egg-shaped {Mo_96_La_8_}^41^ (Figure 3G), (F) egg-shaped {Mo_100_Ce_6_}^42^ (Figure 3G), (G) ellipsoidal {Mo_150_La_2_}^40^ (Figure 3G), (H) ellipsoidal {Mo_134_La_10_}^43^ (Figure 3G), (I) circular-shaped {Mo_92_Ln_9_}^31^ with Ln^III^ = Nd^III^ or Sm^III^ (Figure 3G), (J) circular-shaped {Mo_90_Ln_10_}^31^ with Ln^III^ = La^III^, Ce^III^, or Pr^III^) (Figure 3G), and (K) ellipsoidal half closed {Mo_130_Ce_6_}^32^ (Figure 3H). The open side is shown on the left and the closed side on the right. Coloring code: {MoO_6_}, yellow; {Mo_2_O_11_}, red; {Mo_8_O_35_}, blue with central {MoO_7_}-unit in cyan; {LaO_9_}, green.

**Figure 6:**
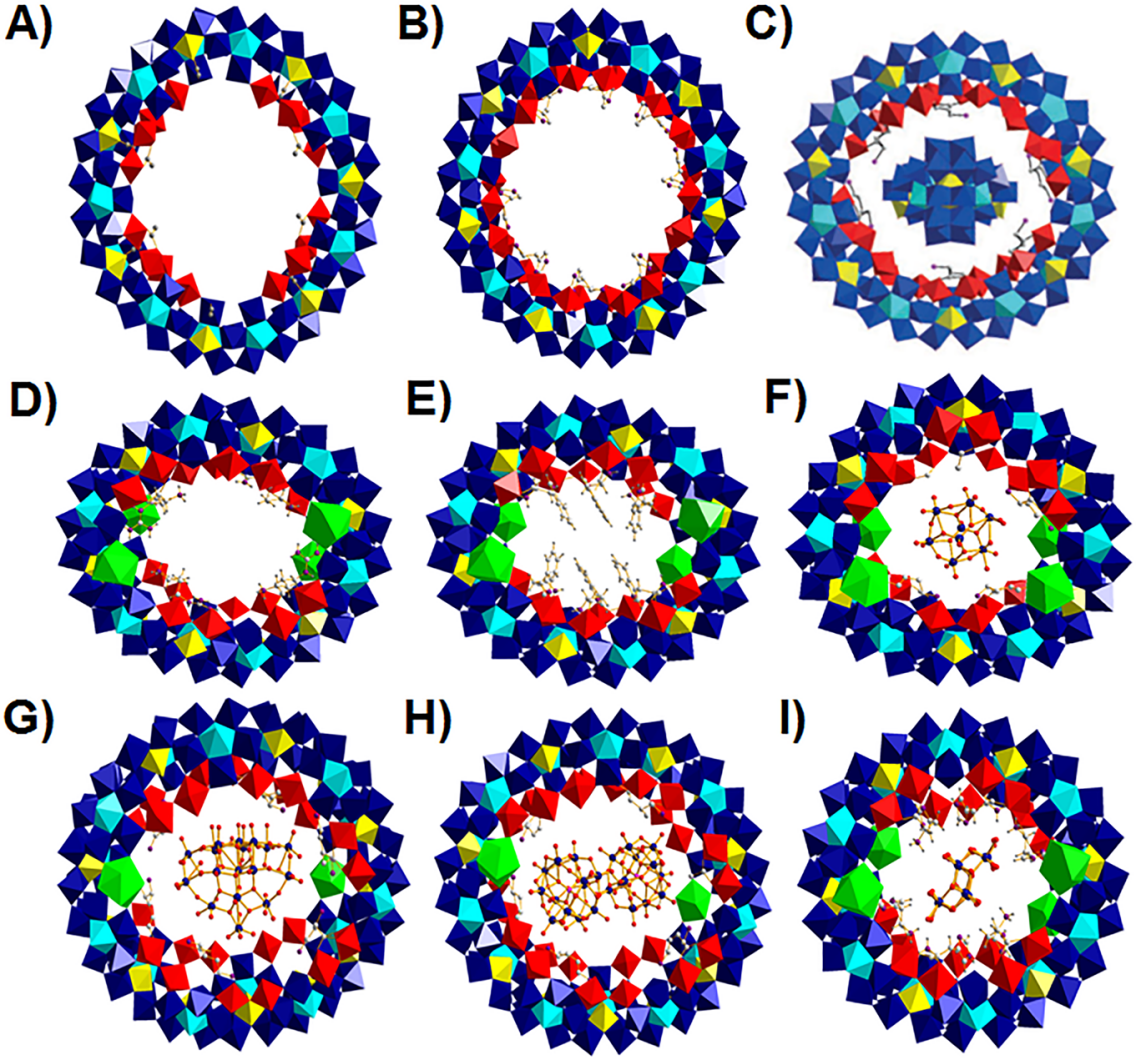
Polyhedral representation of a lacunary hybridized MB, hybridized complete MB, hybridized complete Ln-MB, and templated and hybridized complete Ln-MB ring systems described in this Review starting with (A) six acetates functionalizing {Mo_138_} forming the inorganic–organic hybrid {Mo_138_(acetate)_6_}^20^ (simplified archetype is shown in Figure 3F), (B) the functionalized {Mo_154_} scaffold with eight l-histidines^24^ (Figure 3D), (C) the host–guest complex {Mo_36_}⊂{Mo_150_} functionalized with six l-ornithine^38^ (Figure 3E) [Reprinted with permission from ref (38). Copyright 2019 Wiley-VCH.], (D) the Λ-{Mo_124_Ce_4_}^24^ (Figure 3I) scaffold functionalized with four d-arginines, (E) the Λ-{Mo_124_Ce_4_}^24^ (Figure 3I) scaffold functionalized with six l-tryptophans, (F) the “Japanese rice-ball”-shaped α-{Mo_8_}@{Mo_124_Ce_4_(Orn)_6_}^38^ (Figure 3J), (G) the elliptically shaped {Mo_17_}@{Mo_150_Ce_2_(Orn)_6_}^38^ (Figure 3J), and (H) the elliptically shaped β-{PMo_12_}@{Mo_150_Ce_2_(Orn)_6_}^38^ (Figure 3J) and (I) the Λ-{Mo_124_Ce_4_}^24^ scaffold functionalized with six l-histidines with the chiral {Mo_8_} cluster entrapped in the nanocavity (Figure 3J). Coloring code: {MoO_6_}, yellow; {Mo_2_O_11_}, red; {Mo_8_O_35_}, blue with central {MoO_7_} unit in cyan; {CeO_9_}, green; Mo, blue spheres; C, gray spheres; O, red spheres; N, violet spheres.

Complete MB ring systems (Figure 4A) such as {Mo_154_} are often synthesized at room temperature, with the pH value (typically below 1.4) being the most critical factor due to its strong effect on the nuclearity of the wheel.^1,19^ The complete MB ring system can be transformed into templated complete MB ring systems (Figure 4B) or into lacunary MB ring systems by creating defects (= vacancies caused by a lack of {Mo_2_} building blocks) (Figure 4C). The inclusion of a template was first achieved by synthesizing the unprecedented structure {Mo_248_}, which is often described as a “nanocookie”^22^ and the adjustment of the pH between 1.4 and 4.5 made it possible to synthesize MBs with a different number of defects.^3^ The obtained defect-containing MB wheels are derived from the {Mo_154_}-cluster missing a certain number of {Mo_2_}-units (one unit at pH 1.4 and eight units at pH 4.5). They are specified with {Mo_154–*x*_} (*x* = number of defect sites), with {Mo_138_} being the wheel bearing the largest number of defects (*x* = 16) to date (eight {Mo_2_}-units missing in total) (Figure 5C).^3^ Complete MB ring systems (Figure 3A) can also be organically hybridized to build hybridized complete MB ring systems (Figure 3D). The ligation of {Mo_154_} to eight l-histidine (Figure 6B) molecules represents a good example for organically functionalized MBs.^24^

Organic hybridization reactions can also be used to stabilize anionic templates in the center of MBs via hydrogen bonds resulting in templated and hybridized complete MB ring systems (Figure 4E). An example of such a host–guest complex is {Mo_36_}⊂{Mo_150_(ornithine)_6_} (Figure 6C).^38^ The last representatives of lanthanide-free MB archetypes are lacunary hybridized MB ring systems, which contain defects within their scaffold as well as grafted carboxylic acids onto their remaining {Mo_2_}-units (Figure 4F). Lacunary hybridized MB ring systems were initially realized with the organic-chemical hybridization of a {Mo_138_} cluster leading to the inorganic–organic MB {Mo_138_(acetate)_6_}^20^ (Figure 6A). Figure 4 gives an overview of the main synthesis factors in the assembly of all structural types of MB-based clusters.

### 3.2 Ln-MB Based Nanoclusters

Preventing the integration of {Mo_2_} units ({Mo^VI^_2_O_5_(H_2_O)_2_}^2+^), which contribute to the construction of the inner ring, into the wheel framework by the use of large electrophilic “open-shell” metal centers such as lanthanides (consistently incorporated as trivalent cations) alters the overall charge, molecular shape, and size of these nanostructures compared to the parent {Mo_154_} ring (Figure 5A). The introduction of Ln^III^ ions into MB frameworks always causes a symmetry reduction compared to the highly symmetrical *D*_7*d*_ {Mo_154_} nanocluster (Table 1). As a result, lanthanide ions are often referred to as “symmetry breakers” in the context of MBs. Figure 3 includes all lanthanide-containing MB structural archetypes (Figure 3G–J) that can currently be assembled and Table 2 lists all crystal structures of lanthanide-containing molybdenum wheels as of August 2021, including information about the geometry, lanthanide pattern within the wheel, the most critical reaction parameters (such as total reagents and their stoichiometric ratios, pH (if specified), reducing agent/method, temperature, and reaction time), and applied methods to characterize the structure.

**Table 1:** Prominent Representatives of Purely Inorganic Complete Ln-MB Ring Systems Arranged in Descending Symmetry to the Parent MB {Mo_154_}

cluster	lanthanide ions	symmetry	ref
{Mo_154_}		*D*_7*d*_	(19)
{Mo_120_Ln_6_}	La^III^, Ce^III^, Pr^III^	*D*_3_	(25, 37, 40)
{Mo_128_Ln_4_}	Eu^III^	*D*_2_	(44)
{Mo_90_Ln_10_}	Ce^III^	*D*_5*d*_	(31)
[Mo_130_Ln_6_}	Ce^III^	*C*_2*v*_	(32)
{Mo_124_Ln_4_}	Ce^III^	*C*_2_	(24)
{Mo_96_Ln_8_}	La^III^	*C*_2_	(41)
{Mo_100_Ln_6_}	Ce^III^	*C*_2_	(42)
{Mo_134_Ln_10_}	La^III^	*C*_*i*_	(43)
{Mo_122_Ln_5_}	Ce^III^	*C*_s_	(39)

**Table 2:**
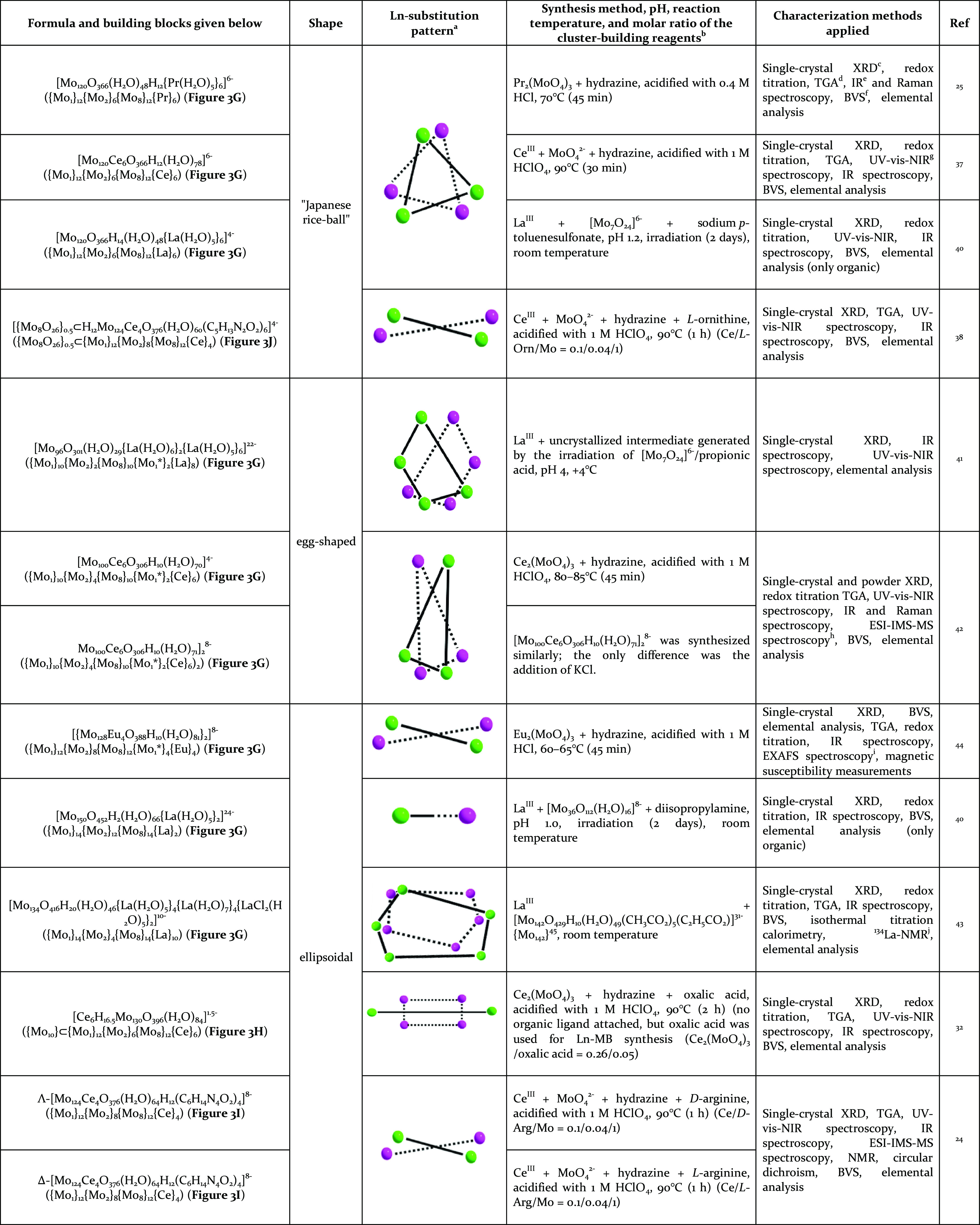

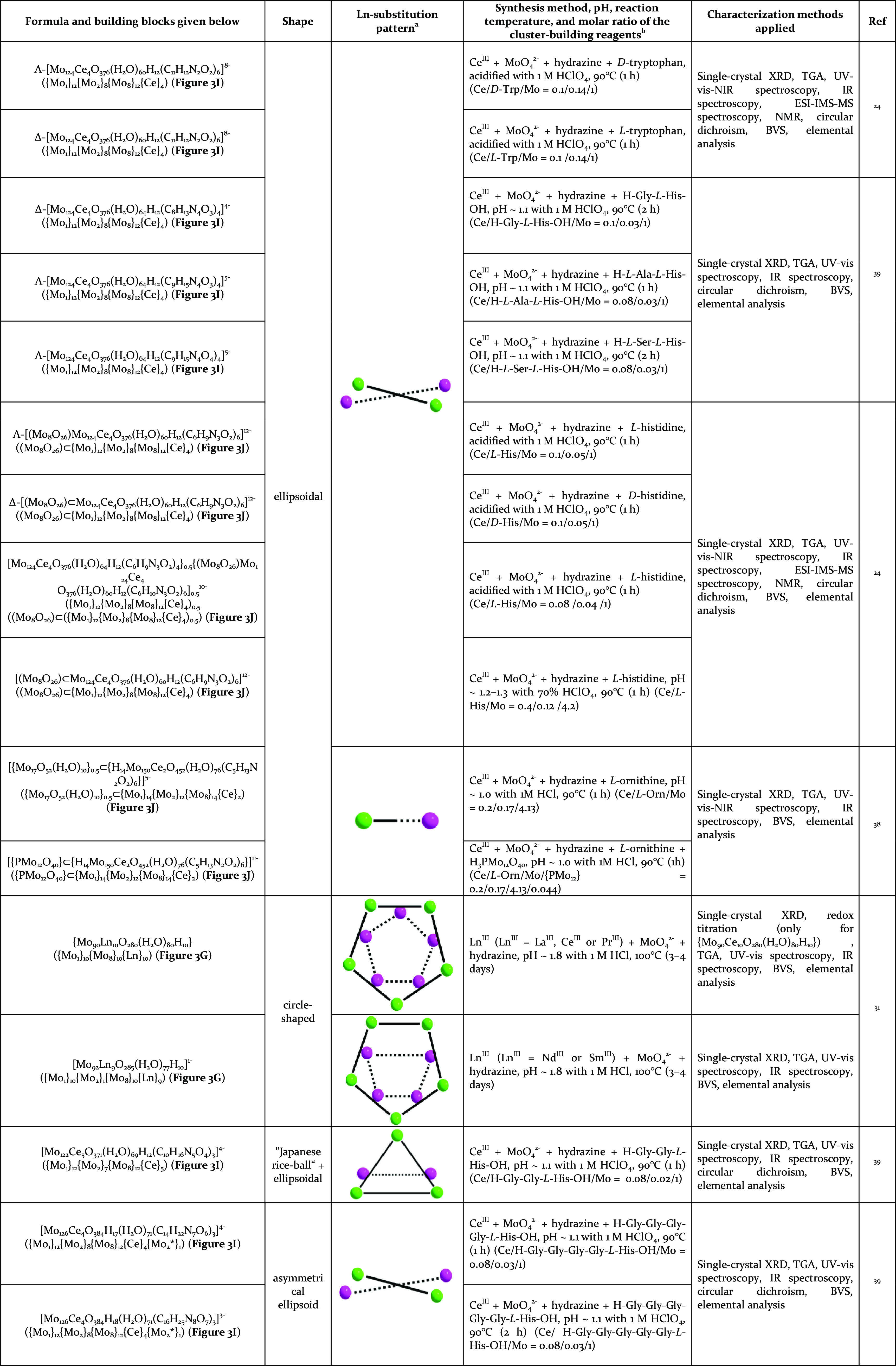
List of All Purely Inorganic and Hybridized Inorganic–Organic Lanthanide-Containing Molybdenum Blue Wheel Crystal Structures According to the CCDC and ICSD Database (August 2021); Ln-MBs Are Listed Based on Their Shape, Starting with the “Japanese Rice-Ball”

a The lanthanide substitution pattern of the respective wheel-type molybdenum blue is shown graphically. The lanthanide ions on the common inner rim of the wheel are shown in one color (green and pink). Important notes regarding the synthesis and characterization of the respective wheel are also indicated.

b As the amount of organic ligand used has a significant influence on the final structure, the molar ratio of the main cluster-building reagents is indicated when an amino acid, peptide, or (di)carboxylic acid was used in the chemical synthesis.

c X-ray diffraction.

d Thermogravimetric analyses.

e Infrared spectroscopy.

f Bond valence sum.

g Ultraviolet–visible-near-infrared spectroscopy.

h Electrospray ionization-ion mobility spectrometry–mass spectrometry.

i Extended X-ray absorption fine structure spectroscopy.

j Lanthan-nuclear magnetic resonance spectroscopy. Building blocks marked with an asterisk (*) indicate an insertion between two {Mo_8_} building blocks.

#### 3.2.1 Synthesis of Purely Inorganic Ln-MBs

##### 3.2.1.1 ″Japanese Rice-Ball”-, Egg-, Ellipsoid-, and Circular-Shaped Ln-MBs without Template Clusters

The “Japanese rice-ball”-shaped wheels {Mo_120_Ln_6_}^25,37,40^ are complete Ln-MB ring systems (Figure 3G and 5D), which exist with Pr^III^, Ce^III^, and La^III^ ions. The Pr^III^- and Ce^III^-containing “Japanese rice-ball”-shaped Ln-MBs were synthesized in a similar manner, but a significantly different synthetic protocol was used for the La^III^-containing cluster. For {Mo_120_Pr_6_}, Pr_2_(MoO_4_)_3_ was first produced by combining a Na_2_MoO_4_ solution with a Pr(NO_3_)_3_ solution. The obtained praseodymium molybdate Pr_2_(MoO_4_)_3_ was then suspended in an acidic solution, reduced, and heated at 70° for 45 min.^25^ For the synthesis of the isostructural cluster {Mo_120_Ce_6_}, no Ce_2_(MoO_4_)_3_ was preformed. However, the synthesis was also performed at a rather high temperature (90 °C for 30 min) in an acidic and reduced medium immediately after all starting materials were produced, combined and had formed a cloudy suspension.^37^ A [NH_4_]_6_[Mo_7_O_24_] solution was photochemically reduced for 2 days at room temperature to get {Mo_120_La_6_}.^40^ All three “Japanese rice-ball”-shaped Ln-MBs crystallize within 1–2 weeks at room temperature (see Table 2 for more details), whereby the lanthanide substitution pattern was built up in a one-pot synthesis and is therefore less controllable.

A complete Ln-MB ring system (Figure 3G) with the shape of an “egg” was first realized through the synthesis of {Mo_96_La_8_} (Figure 5E), in which a lacunary MB with an ambiguous number of defects was first dissolved and then treated with La^III^ ions (see Table 2 for more details).^41^ This “egg formation” was enabled by a more predictable synthesis procedure using a two-step process. An MB containing defects was preformed in the first step, thus allowing 4f metals to react at specific positions within the wheel in the second step. The lacunary MB used in this synthesis was generated as a precipitate by reducing an acidified (pH 3.9–4.1) [Mo_7_O_24_]^6–^-containing solution photochemically for 10 days at a temperature between 10 and 15 °C leading to crystals of {Mo_96_La_8_} in 1 week at 4 °C. Notably, in a one-pot synthesis, the nanosized complete Ln-MB ring system (Figure 3G) {Mo_100_Ce_6_}^42^ (Figure 5F) was generated which also exhibits the shape of an “egg”. {Mo_100_Ce_6_} was assembled by suspending a precipitate of Ce_2_(MoO_4_)_3_ in an acidic solution followed by reducing and heating at 80–85 °C for 1 h. The synthesis protocol of {Mo_100_Ce_6_} differs from that of {Mo_120_Pr_6_} in that {Mo_100_Ce_6_} was synthesized in a 10-fold higher concentration and an ∼4.5-fold lower acid concentration.

**Figure 7:**
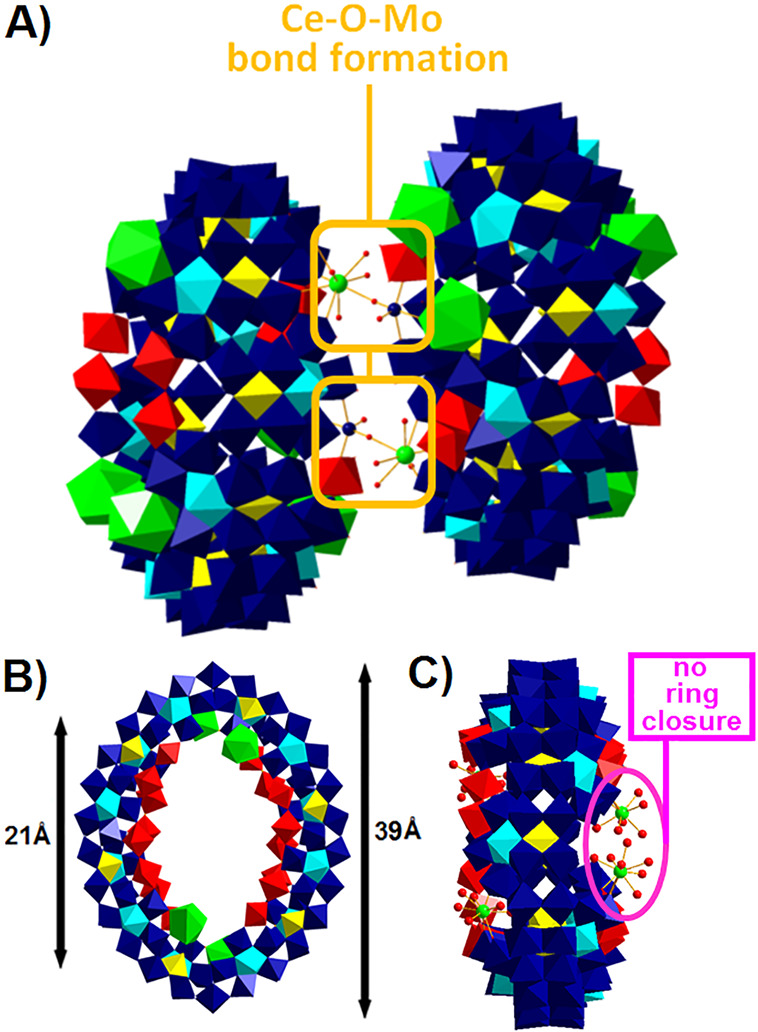
Polyhedral and ball-and-stick representation of the (A) dimer {Mo_100_Ce_6_}_2_^42^ (Figure 3G) and (B) ellipsoidal cluster {Mo_128_Eu_4_}^44^ (Figure 3G) (with a side view shown in (C)). Coloring code: {MoO_6_}, yellow; {Mo_2_O_11_}, red; {Mo_8_O_35_}, blue with central {MoO_7_}-unit in cyan; {CeO_9_} and {EuO_9_}, green; Mo, blue spheres; Ce, green spheres; O, red spheres.

Elliptically shaped Ln-MB scaffolds belonging to complete Ln-MB ring systems (Figure 3G) were assembled for the first time in a one-pot approach through the use of Eu^III^ ions, which have smaller ionic radii than those of Ce^III^ and Pr^III^ ions. For the assembly of {Mo_128_Eu_4_} a precipitate of Eu_2_(MoO_4_)_3_ was suspended in an acidic solution and then reduced with hydrazine and heated at 60–65 °C for 45 min (see Table 2 for more details). The elliptical wheel {Mo_128_Eu_4_} (Figure 7B) led to the crystallization of the dimer {Mo_256_Eu_8_} after 2 weeks.^44^ Remarkably, the self-assembly of the complete Ln-MB ring system (Figure 3G) {Mo_150_La_2_} (Figure 5G) with the shape of an ellipsoid was obtained in a photochemical reduction (irradiation for 2 days) of an acidified (pH 1.0) La^III^-containing solution with the IPOMo {Mo_36_} (Figure 1).^40^ A two-step reaction in which Ln^III^ ions react in the vacancies of preformed wheels can also be applied to synthesize ellipsoidal structures.^43^ This has been realized with the synthesis of the highly Ln-doped complete Ln-MB ring system (Figure 3G) {Mo_134_La_10_} (Figure 5H).^43^ By treating the preformed wheel {Mo_142_}^45^ with La^III^ ions in a NaCl-solution without acidification at room temperature, crystals of {Mo_134_La_10_} form at 4 °C after 2 weeks.

**Figure 8:**
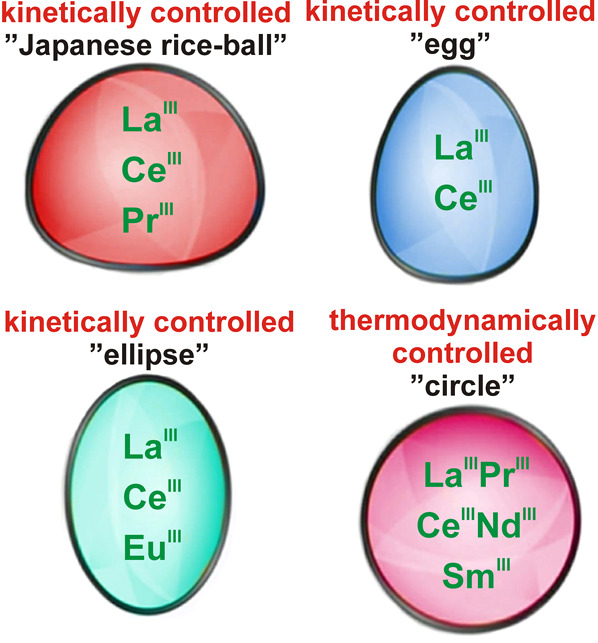
Four basic shapes of Ln-MBs. The “Japanese rice-ball“- (existing in the structural types shown in Figure 3G, I, and J), egg- (existing in the structural type shown in Figure 3G), and ellipsoidal-shaped Ln-MBs (existing in the structural types shown in Figure 3G–J) are all assembled in kinetically controlled syntheses; however, the circular-shaped Ln-MBs (existing in the structural type shown in Figure 3G) are synthesized in thermodynamically controlled reactions. The lanthanides that can be incorporated in the corresponding cluster types are indicated in the center of the shapes.

The “Japanese rice-ball”-, egg-, and ellipsoid-shaped Ln-MBs are all typically synthesized at temperatures 60-90 °C for 30−45min (in a one-pot approach). A fourth type of complete Ln-MB ring systems (Figure 3G), the circular-shaped {Mo_92_Ln_9_} (Figure 5I) and {Mo_90_Ln_10_} (Figure 5J) archetypes, can be generated when the synthesis temperature is increased to 100 °C for up to 4 days accompanied by controlled evaporation of the solvent (see Table 2 for more details). This changes the reaction control from kinetic to thermodynamic control (Figure 8), which is the key step in assembling these circular-shaped Ln-MBs.^31^

##### 3.2.1.2 Ellipsoid-Shaped Ln-MBs with Template Clusters

The incorporation of a template cluster in an Ln-MB can be achieved without hybridizing the scaffold. This was accomplished with the assistance of oxalic acid, a dicarboxylic acid, which acts as a structure-directing ligand without attaching to {Mo_2_}-units to form templated complete Ln-MB ring systems (Figure 3H). This was discovered in the synthesis of {Mo_130_Ce_6_} (Figure 5K), which was assembled by suspending Ce_2_(MoO_4_)_3_ in an acidified aqueous solution, adding oxalic acid and heating to 90 °C for 2 h (see Table 2 for more details). The crucial variable for the assembly of {Mo_130_Ce_6_} appears to be a structural ligand (in this case oxalic acid) in the proper ratio to the molybdate, which does not appear in the final structure but more likely, decomposes during synthesis. This hypothesis is based on the fact that oxalic acid decomposes in an acidic environment at 80 °C.^46^ Single crystals suitable for X-ray diffraction were obtained at room temperature after 2 weeks.

#### 3.2.2 Structural Description of Purely Inorganic Ln-MBs

##### 3.2.2.1 ″Japanese Rice-Ball”-, Egg-, Ellipsoid-, and Circular-Shaped Ln-MBs without Template Clusters

The “Japanese rice-ball”-shaped Ln-MBs are composed of 12 {Mo_1_}, 6 {Mo_2_}, 12 {Mo_8_}, and 6 {Ln} building units (Table 2). Six {Mo_2_}^2+^ groups are replaced by smaller Ln^III^ ions (Ln^III^*=* La^III^, Ce^III^, or Pr^III^; according to reported crystal structures^25,31,42−44^). The average size of the integrated {LnO_9_} in the inner ring (O–Ln^III^–O) is 4.6 Å, while the corner-sharing {Mo_2_} units (O–Mo–O–Mo-O) are 7.3 Å, forcing the cluster into a “more contracted” structure exhibiting an irregular ring-shape and a lower symmetry (*D*_3_)^25^ compared to that of the ideal circular parent structure {Mo_154_}^19^ (*D*_7d_ point group) (Figure 5A). All lanthanide centers on both the upper and lower surfaces of {Mo_120_Ln_6_}^25,37,40^ exhibit a tricapped trigonal prismatic coordination sphere (Figure 2B). “Japanese rice-ball”-shaped Ln-MB clusters reported to date exhibit an inner and outer diameter of ∼17 and ∼31 Å, respectively, and their total charges varies between −4 and −6 (see Table 2).

The egg-shaped Ln-MB {Mo_96_La_8_} (assembled in a two-step approach) consists of 10 {Mo_1_}, 2 {Mo_2_}, 10 {Mo_8_}, 2 {Mo_1_*} (building blocks marked with an asterisk indicate an insertion between two {Mo_8_} building blocks), and 8 {La} building units (Table 2) and exhibits an inner and outer diameter of ∼14 and ∼30 Å, respectively, with four lanthanide ions and one {Mo_2_} unit coordinated in each inner rim (Figure 5E).^41^ In comparison to the “Japanese rice-ball″-shaped scaffolds {Mo_120_Ln_6_} (Ln^III^*=* La^III^, Ce^III^, or Pr^III^) (Figure 5D), which were synthesized in a one-pot reaction, the “egg” {Mo_96_La_8_} is smaller and has a less uniform lanthanide pattern. {Mo_96_La_8_} was characterized as a heteropolyacid with a relatively high negative charge of minus 22 due to the lack of any ammonium counter-cations, as verified by elemental analysis (see Table 2). In the egg-shaped structure {Mo_100_Ce_6_}, which is composed of 10 {Mo_1_}, 4 {Mo_2_}, 10 {Mo_8_}, 2 {Mo_1_*}, and 6 {Ln} building units (Table 2), six Ce^III^ ions are coordinated to the Ln-MB wheel, causing a contraction and consequently forcing the cluster to take on the shape of an egg with an inner and outer ring diameter of ∼16 and ∼31 Å, respectively, very close to that of the “Japanese rice-ball” (Figure 5F).^42^ The lanthanide substitution pattern, however, differs from that of the “Japanese rice-ball”, especially in the distribution of the 4f-metals within the scaffold. The six Ce^III^ ions form two triangles (see Table 2), one located on the upper rim and the other one on the lower rim of the wheel. In each triangle, two Ce^III^ ions are located adjacent to each other at one end of the wheel, whereas the third Ce^III^ ion is situated at the opposite end. Without the addition of KCl, this third Ce^III^ ion coordinates to a neighbor wheel via a Ce–O–Mo bond, leading to the formation of the dimer {Mo_100_Ce_6_}_2_^42^ (Figure 7A).^42^ The monomer {Mo_100_Ce_6_} exhibits a negative charge of four minus and the dimer {Mo_100_Ce_6_}_2_ of minus eight (see Table 2).

The Ln-MB {Mo_128_Eu_4_}, exhibiting a negative charge of minus eight, is assembled under very similar conditions as {Mo_120_Pr_6_} and {Mo_100_Ce_6_}, however, the substitution of Pr^III^ or Ce^III^ by Eu^III^ results in a significantly larger ellipsoidal-shaped cluster with 12 {Mo_1_}, 8 {Mo_2_}, 12 {Mo_8_}, 4 {Mo_1_*}, and 4 {Eu}-building units and an inner and outer ring diameter of ∼21 and ∼39 Å (Table 2), respectively, rather than a “Japanese rice-ball” or egg-shaped cluster.^44^ Notably, Eu^III^ ions not only reduce the *D*_7*d*_ symmetry of the parent {Mo_154_} wheel to *D*_2_ (Table 1) but also prevent the parent-ring closure (Figure 7C). The ellipsoidal wheel {Mo_150_La_2_} is composed of 14 {Mo_1_}, 12 {Mo_2_}, 14 {Mo_8_}, and 2 {La} building units with an inner and outer diameter of ∼22 and ∼38 Å, respectively, and the second largest Ln-MB cluster to date with the greatest negative charge (−24) (Table 2).^40^ Two distorted tricapped-trigonal-prismatic La^III^ centers (green polyhedra) are incorporated into the ring structure instead of two {Mo_2_}-units (red polyhedra) (Figure 5G) (see Table 2 for more details). {Mo_134_La_10_}, also an ellipsoidal-shaped Ln-MB, is currently the largest Ln-MB wheel with an inner and outer diameter of ∼21 and ∼39 Å, respectively. It is composed of 14 {Mo_1_}, 4 {Mo_2_}, 14 {Mo_8_}, and 10 {Ln} building units with five lanthanide ions and two {Mo_2_}units coordinated in each inner rim (Figure 5H). Interestingly, in the synthesis of {Mo_134_La_10_}, when treating the six “defect pockets” (=6 missing {Mo_2_} units) of the MB {Mo_142_}^45^ (={Mo_154–12_}) with La^III^ ions, not only six lanthanide ions are incorporated in these vacant sites but four {Mo_2_} units are also substituted by La^III^ ions, causing the wheel shape to change from circular to ellipsoid. This shape modification was confirmed by isothermal titration calorimetry.^43^

Intriguingly, Ln-MBs are able to assemble with all {Mo_2_}-units of the inner rim being replaced by lanthanide ions.^31^ However, due to the lanthanide-induced contraction of the wheel structure, the replacement of all inner-rim {Mo_2_}-units was only feasible with the large ions La^III^, Ce^III^, and Pr^III^, yielding the first (and currently only) charge-neutral circular-shaped wheel {Mo_90_Ln_10_}^31^ (Ln^III^ = La^III^, Ce^III^, or Pr^III^) (Figure 5J). {Mo_90_Ln_10_} consists of 10 {Mo_1_}-, 10 {Mo_8_}-, and 10 {Ln}-building units (Table 2) and exhibits an inner and outer diameter of ∼10 Å and ∼26 Å, respectively (Figure 5J). With Nd^III^ and Sm^III^, which belong to the later lanthanides and therefore have a smaller radius, the scaffold {Mo_92_Ln_9_}^31^ (Ln^III^ = Nd^III^ or Sm^III^) with a charge of minus one was obtained (Figure 5I) in which one {Mo_2_} building block unit is preserved in the wheel. {Mo_92_Ln_9_} is composed of 10 {Mo_1_}-, 1 {Mo_2_}-, 10 {Mo_8_}-, and 9 {Ln}-building units (Table 2). The replacement of all inner-rim {Mo_2_}-units could not be accomplished in {Mo_92_Ln_9_}^31^ as the wheel contraction would become too large (radii: Sm^III^ < Nd^III^ < Pr^III^ < Ce^III^ < La^III^ < {Mo_2_}^2+^). The lanthanide substitution pattern of {Mo_90_Ln_10_} and {Mo_92_Ln_9_} does not induce a structural deformation compared to the origin cluster {Mo_154_}, in contrast to {Mo_120_Pr_6_},^25^ {Mo_100_Ce_6_},^42^ {Mo_96_La_8_},^41^ and {Mo_128_Eu_4_}.^44^

##### 3.2.2.2 Ellipsoid-Shaped Ln-MBs with Template Clusters

The ellipsoidal templated wheel {Mo_130_Ce_6_} is made up of 12 {Mo_1_}, 6 {Mo_2_}, 12 {Mo_8_}, and 6 {Ce} building units with an additionally enclosed {Mo_10_} fragment and exhibits an inner and outer diameter of ∼18 and ∼33 Å, respectively (Figure 5K). {Mo_130_Ce_6_} is composed of a {Mo_120_Ce_6_} ring that is half-closed by the polyoxomolybdate “cap” {Mo_10_}.

Fascinatingly, only the use of oxalic acid enabled the trapping of a {Mo_10_} fragment on just one side of the ellipsoidal {Mo_120_Ce_6_} ring without hybridizing the {Mo_2_} units organically (Figure 5K).^32^ This entrapment, which is the only example of a semiclosed Ln-MB to date, probably occurs through either supramolecular interactions, such as hydrogen bonding, or oxalic acid serving as a transient anion template. In {Mo_130_Ce_6_}, four Ce^III^ ions are coordinated on the open side of the ellipsoidal scaffold, and the remaining two Ce^III^ ions are incorporated on the closed side containing the {Mo_10_} cap (Figure 5K).

##### 3.2.2.3 The Four Basic Shapes of Ln-MBs: Which Ln^III^ Ion Is Responsible for Which Shape?

La^III^, the lanthanide with the largest radius, was built into all four basic forms (Figure 8) either photochemically and under moderate reaction conditions,^40^ by occupying defect sites in preformed lacunary MBs,^43^ or through a thermodynamically controlled self-assembly^31^ (see Table 2). The capability of La^III^ ions to be introduced into MBs under mild reaction conditions (syntheses at room temperature) indicates that La^III^ ions may be more reactive in MB systems than the rest of the lanthanide family. All four basic forms (Figure 8) and all Ln-MB ring systems (Figure 3G–J) are synthesizable with Ce^III^ ions, which leads to the assumption that Ce^III^ ions are of ideal size and reactivity. Ce^III^ containing Ln-MB reaction systems are likely to be the most versatile ones, which explain the relatively high number of Ce^III^ containing Ln-MBs that have been reported (20 as of August 2021; see Table 2).

Pr^III^ ions, the third-largest lanthanides, are currently only known in “Japanese rice-ball”- and circular-shaped Ln-MBs.^25,31^ The latter shape could hitherto only be assembled thermodynamically. The smaller lanthanide ions Nd^III^ and Sm^III^ have so far only been incorporated into circular-shaped Ln-MBs in thermodynamically controlled Ln-MB synthesis approaches.^31^ Due to their ion size, Nd^III^ and Sm^III^ seem reluctant to be incorporated into “Japanese rice-balls” or “eggs”, which are typically formed in kinetically controlled synthesis approaches. Surprisingly, it is feasible to incorporate Eu^III^, the smallest lanthanide ion so far (which one would only expect to be incorporated thermodynamically into a circular-shaped Ln-MB ring due its size) into an ellipsoidal Ln-MB through a kinetically controlled reaction.^44^ However, the incorporation of Eu^III^ into an ellipsoidal Ln-MB prevented ring closure of the inner rim (Figure 7C), which is most likely owing to its relatively small size. An open inner rim in an Ln-MB is only known for Eu^III^ ions.

The size of a lanthanide determines whether it can replace a {Mo_2_} unit in MBs. The smallest lanthanide ion integrated in the inner rim of MBs and identified by X-ray structure analysis is Eu^III^.^44^ This suggests that the slightly larger lanthanide ions Nd^III^ and Sm^III^ can likewise serve as building units in kinetically controlled self-assemblies of Ln-MBs. The lanthanide ions from Gd^III^ to Lu^III^ appear to be too small to function as building units in Ln-MBs.

**Figure 9:**
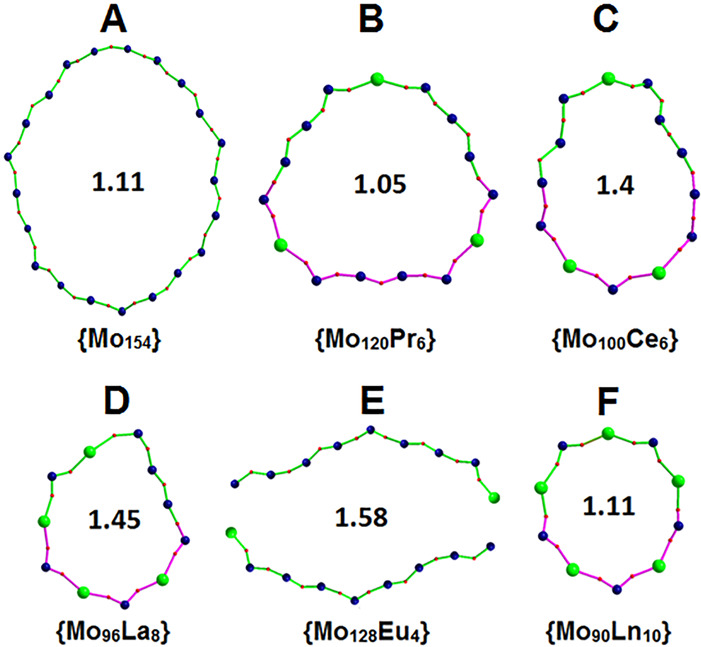
Ball-and-stick representation of the rim skeleton of the (A) circular-shaped {Mo_154_}^19^ (Figure 3A), (B) “Japanese rice-ball″-shaped {Mo_120_Pr_6_}^25^ (Figure 3G), (C) egg-shaped {Mo_100_Ce_6_}^42^ (Figure 3G), (D) egg-shaped {Mo_96_La_8_}^41^ (Figure 3G), (E) ellipsoidal-shaped {Mo_128_Eu_4_}^44^ (Figure 3G), and (F) circular-shaped {Mo_90_Ln_10_}^31^ (Ln^III^ = La^III^, Ce^III^, or Pr^III^) (Figure 3G). The aspect ratio (longest/shortest distance of the inner cavity) is indicated in the center of the wheels. The symmetry-related Ln^III^ ions are omitted for clarity. The metal–oxygen bonds of asymmetrical cavities are highlighted two-colored (green and pink). Coloring code: Mo, blue; Ln, green; O, red.

When incorporated in MBs lanthanide ions generate significant deformations relative to the circular parent cluster {Mo_154_}, resulting in the main basic shapes (“Japanese rice-ball”, “egg”, and ellipsoidal forms) (Figure 8). However, if the degree of replacement of the {Mo_2_} units by Ln^III^ ions is very high (9 or 10 Ln^III^ ions per molecule), the resulting shape of the Ln-MB is nearly identical to that of {Mo_154_}. In order to visualize the effect of the integration site of built-in lanthanides generating deformations and to compare them directly, the cavity skeletons of the circular-shaped {Mo_90_Ln_10_}^31^ (Ln^III^ = La^III^, Ce^III^, or Pr^III^), the “Japanese rice-ball” {Mo_120_Pr_6_},^25^ the egg-shaped {Mo_100_Ce_6_}^42^ and {Mo_96_La_8_},^41^ as well as the ellipsoidal {Mo_128_Eu_4_}^44^ are illustrated in Figure 9. Due to the even distribution of both the Pr^III^ ions within the inner surface of {Mo_120_Pr_6_}^25^ and the Ln^III^ ions in {Mo_90_Ln_10_}^31^ as well as the antipodal location of the Eu^III^ ions in {Mo_128_Eu_4_},^44^ these wheels show a more regular structure than the remaining wheels in Figure 9. In contrast, the uneven distribution of three Ce^III^ ions in the inner rim of {Mo_100_Ce_6_}^42^ forms a triangle in which two Ce^III^ ions are located adjacent to each other (Ce–O–Mo–O–Ce) while the third ion is positioned at the opposite side of the cavity. Also, the uneven lanthanide ion distribution in {Mo_96_La_8_},^41^ where all four La^III^ ions are asymmetrically positioned and connected to each other via O–Mo–O bridges results in an irregular rather than regular structure. Depending on the nature of the lanthanide, a specific replacement of {Mo_2_}-units can be accomplished, allowing for curvature tuning.

#### 3.2.3 Synthesis of Hybridized Ln-MBs

As of August 2021, there are 11 crystal structures of hybridized complete Ln-MB ring systems (Figure 3I) and 7 templated and hybridized complete Ln-MB ring systems (Figure 3J) reported (Table 2).

Wheel-shaped clusters have a chaotropic character, due to the large molecular surface with a relatively low charge density. Recent findings have demonstrated that formation in aqueous solutions of adducts with organic molecules (e.g., cyclodextrins), which originates from the general chaotropic nature of POMs, dramatically increases the kinetics of MB wheels and may open up new pathways in Ln-MB synthesis.^47^

##### 3.2.3.1 Ln-MBs without Template Clusters

The setting of synthesis parameters is essential for the design of hybridized complete Ln-MB ring systems (Figure 3I). The pH value should be below 1.4 and the synthesis temperature about 90 °C for 1–2 h. To generate single-crystals for X-ray structure analysis, the lanthanide/ligand/molybdate ratio (see Table 2) in the synthesis must be quite precise in order to prevent precipitation and poor-quality crystals.

**Scheme 1:**
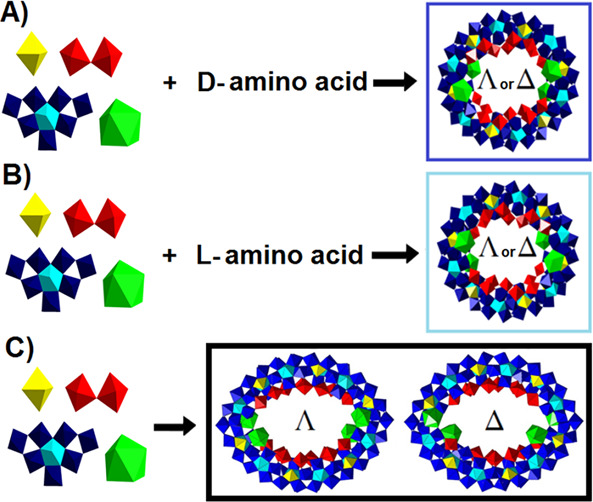
Schematic Representation of the Stereoselective Synthesis of Chiral Ln-MBs through the Use of Chiral Amino Acids (A) d-Histidine results in the Δ-{Mo_124_Ce_4_(d-histidine)_6_} enantiomer (Figure 3I), whereas d-tryptophan and d-arginine yield the Λ-{Mo_124_Ce_4_(d-tryptophan)_4_} and Λ-{Mo_124_Ce_4_(d-arginine)_4_} (Figure 3I), enantiomers.^24^ (B) l-Histidine results in the Λ-{Mo_124_Ce_4_(l-histidine)_6_} enantiomer (Figure 3I), whereas l-tryptophan and l-arginine yield the Δ-{Mo_124_Ce_4_(l-tryptophan)_4_} and Δ-{Mo_124_Ce_4_(l-arginine)_4_} (Figure 3I), enantiomers.^24^ (C) Without the addition of a chiral amino acid, {Mo_126_Ce_4_}^24^ (Figure 3G) [Reprinted from ref (24). Copyright 2018 American Chemical Society.] is obtained as a racemate. Coloring code: {MoO_6_}, yellow; {Mo_2_O_11_}, red; {Mo_8_O_35_}, blue with central {MoO_7_}-unit in cyan; {CeO_9_}, green. Λ and Δ represent right-handed and left-handed propeller, respectively and organic ligands in the final structure are omitted for clarity.

When lanthanide ions are introduced into MBs, their symmetry is invariably reduced in comparison to the parent cluster {Mo_154_} (Table 1), resulting in chiral racemic Ln-MB arrangements.^24^ Enantiopure hybridized complete Ln-MB ring systems can be produced if a chiral amino acid is present in the self-assembly process of Ln-MBs. Since Ln^III^ ions and chiral amino acids exert a synergistic effect in acidified solutions of reduced molybdates, the selective assembly of a series of enantiopure Ln-MBs with the formula Δ/Λ-{Mo_124_Ce_4_(amino acid)_4-or-6_} (Scheme 1) was enabled.^24^ A left-handed propeller-like configuration is represented by the symbol Δ, whereas a right-handed propeller-like arrangement is represented by Λ.

Δ/Λ-{Mo_124_Ce_4_(amino acid)_4-or-6_} are chiral hybridized complete Ln-MB ring systems (Figure 3I) synthesized in a one-pot approach as follows: an acidified aqueous solution containing all main starting materials (CeCl_3_, Na_2_MoO_4_ and the respective proteinogenic amino acid) (see Table 2 for more details) was reduced and heated at 90 °C for 1 h. Crystals suitable for X-ray diffraction were so far only obtained with the chiral arginine (after 1 week at room temperature), and tryptophan (after 5 weeks at room temperature) (Figure 6D and E).

The discovery that single enantiopure proteinogenic amino acids significantly affect the self-assembly and stereochemistry of Ln-MBs prompted researchers to investigate enantiopure oligopeptides, which extended the number of hybridized complete Ln-MB ring systems (Figure 3I) by six structures.^39^ In a one-pot synthesis, an acidified aqueous solution containing all main starting materials (CeCl_3_, Na_2_MoO_4_ and the respective histidine-terminated oligopeptide) (see Table 2 for quantity ratios) was reduced and heated at 90 °C for 2 h. Crystals of {Mo_122_Ce_4_(oligopeptide)_3_}, {Mo_124_Ce_4_(oligopeptide)_4_}, and {Mo_126_Ce_4_(oligopeptide)_2_} were obtained at room temperature after two to 8 weeks.^39^ The reaction solution was occasionally heated for a longer period of time (2 h) than in the case for the hybridization of Ln-MBs with proteinogenic amino acids (1 h).

##### 3.2.3.2 Ln-MBs with Template Clusters

To date, Ln-MBs with template clusters have only been assembled with the assistance of organic ligands.^24,32,38^ It is quite difficult to ascertain which ligand will be able to enclose a polyoxometalate as a template. The only established requirement for the ligand (aside from the carboxyl group for grafting to the {Mo_2_} units) is the presence of a nitrogen-containing pendant group (e.g., histidine, ornithine). In an acidic environment, this nitrogen-containing group gets protonated and could therefore stabilize template clusters within the Ln-MB electrostatically and through hydrogen bonds. The use of chiral proteinogenic amino acids not only enables the organic hybridization of the inner rim of Ln-MBs, resulting in nanosized chiral Ln-MBs, but also the synthesis of chiral templated and hybridized complete Ln-MB ring systems (Figure 3J). For the self-assembly of Δ-{Mo_8_}@{Mo_124_Ce_4_(histidine)_6_} and Λ-{Mo_8_}@{Mo_124_Ce_4_(histidine)_6_}, an acidified aqueous solution containing all of the major starting materials (CeCl_3_, Na_2_MoO_4_, and l-histidine (for the Λ-enantiomer) or d-histidine (for the Δ-enantiomer) was reduced and heated at 90 °C for 1 h leading to suitable crystals for X-ray diffraction after 1 week at room temperature.^24^ Besides the proteinogenic amino acids histidine,^24^ arginine,^24^ and tryptophan,^24^ the nonproteinogenic amino acid l-ornithine (Orn)^38^ also has a template-directing effect in the assembly of Ln-MBs. l-Ornithine enabled the synthesis of the “Japanese rice-ball”-shaped (almost ellipsoidal shaped) α-{Mo_8_}@{Mo_124_Ce_4_(Orn)_6_}^38^ (Figure 6F), the elliptically shaped {Mo_17_}@{Mo_150_Ce_2_(Orn)_6_} (Figure 6G), and β-{PMo_12_}@{Mo_150_Ce_2_(Orn)_6_}^38^ (Figure 6H). The critical synthetic variable in the self-assembly of these three templated and hybridized complete Ln-MB ring systems (Figure 3J) is the concentration of the lanthanide ion, ligand, and molybdate. For {Mo_17_}@{Mo_150_Ce_2_(Orn)_6_} and β-{PMo_12_}@{Mo_150_Ce_2_(Orn)_6_}, the concentrations of the ornithine and molybdate were ∼3-fold and that of the Ce^III^ ions ∼1.5-fold higher than for the formation of α-{Mo_8_}@{Mo_124_Ce_4_(Orn)_6_} (see Table 2 for details). In α-{Mo_8_}@{Mo_124_Ce_4_(Orn)_6_} and {Mo_17_}@{Mo_150_Ce_2_(Orn)_6_}, the templated polyoxomolybdate was formed in situ, whereby the enclosed template in β-{PMo_12_}@{Mo_150_Ce_2_(Orn)_6_} was a separately added preformed Keggin.^38^ The ornithine and molybdate concentrations were significantly increased most likely to enhance the ligand’s template-directing effect and to enable both the encapsulation and stabilization of a larger negatively charged polyoxomolybdate cluster.

#### 3.2.4 Structural Description of Hybridized Ln-MBs

Remarkably, organic hybridization of {Mo_2_} building blocks in Ln-MB assemblies predominantly yields ellipsoidal-shaped clusters. The hybridization of {Mo_2_} units in the cavity of ellipsoidal Ln-MBs seems to be less restricted by steric hindrances as in “Japanese rice-balls”, “eggs”, and circular-shaped clusters.

##### 3.2.4.1 Structural Description of Ln-MBs without Template Clusters

**Figure 10:**
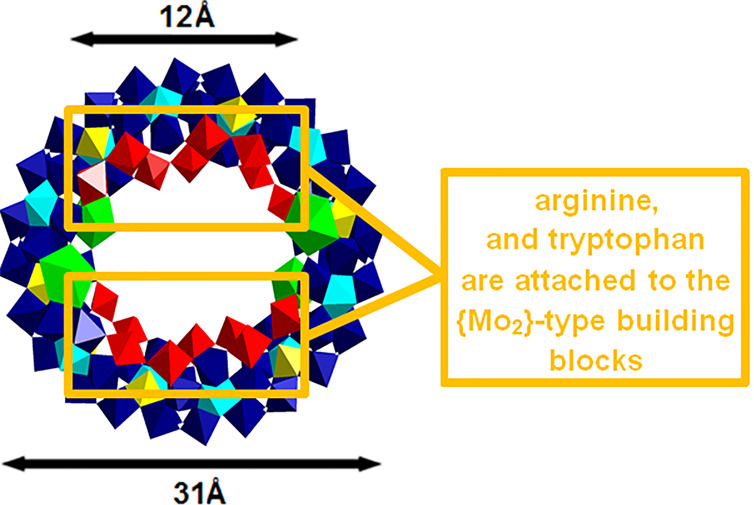
Polyhedral representation of the {Mo_124_Ce_4_}^24^ scaffold of the hybridized, elliptical Ln-MB frameworks (Figure 3I). Coloring code: {MoO_6_}, yellow; {Mo_2_O_11_}, red; {Mo_8_O_35_}, blue with central {MoO_7_}-unit in cyan; {CeO_9_}, green.

All Ln-MBs without template clusters obtained through the hybridization with proteinogenic amino acids are hybridized complete Ln-MB ring systems (Figure 3I) sharing the same elliptical skeleton {Mo_124_Ce_4_}^24^ with either four arginines^24^ (Figure 6D) or six tryptophans^24^ (Figure 6E) grafted to the cluster. The skeleton {Mo_124_Ce_4_} consists of 12 {Mo_1_}, 8 {Mo_2_}, 12 {Mo_8_}, and 4 {Ce} building units (Table 2) and exhibits an inner and outer ring diameter of approximately 12 and 31 Å, respectively (Figure 10). The four Ce^III^ ions built into the scaffold are symmetrically located on the upper and lower rims of the wheel, and the MB-functionalizing amino acids arginine and tryptophan are coordinated to the organically chemically modifiable^23^ {Mo_2_}-type building blocks via their carboxylate groups (Figure 10).

**Scheme 2:**
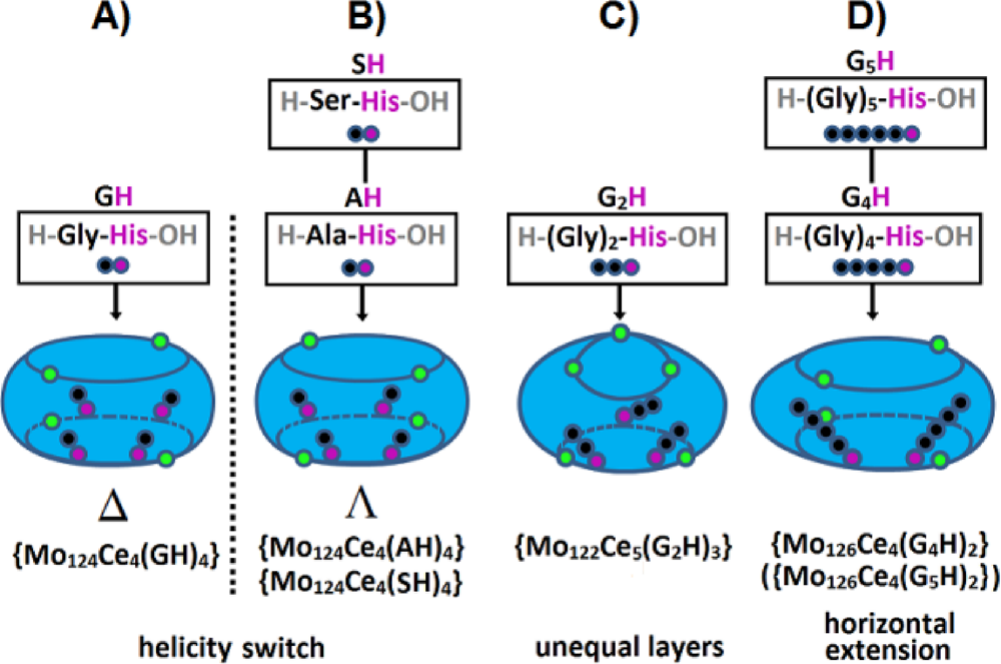
Schematic representation of the histidine-terminated oligopeptide-controlled synthesis of Ln-MBs^39^ The oligopeptides used were (A) H-Gly-l-His-OH (GH) (Figure 3I), (B) H-l-Ala-l-His-OH (AH) (Figure 3I) and H-l-Ser-l-His-OH (SH) (Figure 3I), (C) H-Gly-Gly-l-His-OH (G_2_H) (Figure 3I), and (D) H-Gly-Gly-Gly-Gly-l-His-OH (G_4_H) (Figure 3I) and H-Gly-Gly-Gly-Gly-Gly-l-His-OH (G_5_H) (Figure 3I). The toroidal shapes of the wheels are displayed in blue. The lanthanide ions (Ce^III^) are shown in green; carbon, black; and nitrogen, purple.

Different enantiomers are generated depending on the concentration, type, and configuration of the used amino acid. d- and l-amino acids are capable of producing the Λ and Δ enantiomer in the pure form, the configuration of the amino acid not favoring any specific enantiomer (Scheme 1). In the absence of d- or l-amino acids, only the {Mo_126_Ce_4_} (Scheme 1) wheel is obtained as a racemate, which is structurally similar to {Mo_124_Ce_4_}^24^ (slightly compressed in contrast to {Mo_124_Ce_4_}). Incorporating histidine-terminated oligopeptides into Ln-MB systems revealed that the length and sequence of the peptides markedly influence the shape, Ln-substitution pattern, and stereochemistry of the resulting Ln-MBs.^39^ For instance, if glycine (Scheme 2A) is replaced with serine/alanine (Scheme 2B) in a histidine-based dipeptide, an ellipsoidal Λ-nanowheel is obtained, rather than an ellipsoidal Δ-nanowheel. A very distinct “Japanese rice-ball″-structure is formed, when a histidine-based tripeptide contains two glycines (Scheme 2C). With four glycines in a histidine-based tetrapeptide, a one-sided deformed ellipsoid is constructed that resembles an “egg” (Scheme 2D). X-ray structure analysis showed that all investigated peptides on {Mo_2_} units coordinate exclusively on one side of the Ln-MBs in different stoichiometric ratios (Scheme 2).

**Figure 11:**
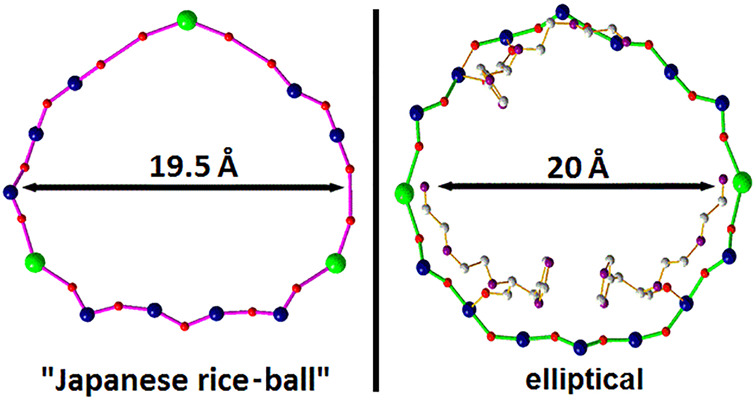
Ball-and-stick representation of the “Japanese rice-ball”- and oligopeptide-hybridized elliptical-shaped cavities of {Mo_122_Ce_5_}.^39^ The metal–oxygen bonds of the two different cavities are highlighted in different colors (green and pink). Coloring code: Mo, blue spheres; Ce, green spheres; C, gray spheres; O, red spheres; N, violet spheres.

The dipeptides H-Gly-l-His-OH (GH), H-l-Ala-l-His-OH (AH), and H-l-Ser-l-His-OH (SH) are attached to the ellipsoidal {Mo_124_Ce_4_}^39^ scaffold, where GH mediates the self-assembly of the Δ-{Mo_124_Ce_4_} enantiomer, and AH and SH that of the Λ-{Mo_124_Ce_4_} enantiomer. Thus, the helicity of the chiral {Mo_124_Ce_4_}-Ln-MBs depends on the N-terminal rather than the C-terminal residue of the oligopeptide. This allows an elegant switch from the Δ to the Λ enantiomer by just altering the amino acid at the N-terminus without changing the stereochemistry of the entire peptide. The tripeptide H-Gly-Gly-l-His-OH mediates the self-assembly of the wheel-skeleton {Mo_122_Ce_5_}^39^ and binds to it three times (Scheme 2C). {Mo_122_Ce_5_} is an unprecedented Ln-MB scaffold containing two differently shaped cavities in the same Ln-MB (“Japanese rice-ball”- and elliptical-shaped cavity, Figure 11) and an odd number of lanthanide ions. The pentapeptide H-Gly-Gly-Gly-Gly-l-His-OH and hexapeptide H-Gly-Gly-Gly-Gly-Gly-l-His-OH mediate racemic Ln-MB mixtures, comprising the asymmetric ellipsoidal scaffold {Mo_126_Ce_4_}^39^ (Scheme 2D). Since the oligopeptides differ in length and sequence, they form different numbers of hydrogen bonds when introduced into Ln-MBs, resulting in a variety of distinct structural characteristics.

##### 3.2.4.2 Structural Description of Ln-MBs with Template Clusters

Notably, only the functionalization of {Mo_124_Ce_4_} scaffold with the proteinogenic amino acids l-/d-histidine transforms the nanocavity into a reaction tube, which can be described as a confined molecular reaction vessel, wherein the in situ assembled, chiral metal cluster {Mo_8_} (structurally related to γ-[Mo_8_O_26_]^4−^^48^) is embedded.^24^ The inclusion of the chiral cluster {Mo_8_} into {Mo_124_Ce_4_} scaffold results in the formation of the supramolecular guest@host assembly {Mo_8_}@{Mo_124_Ce_4_(histidine)_6_}^24^ (Figure 6I). The orientation of the introduced {Mo_8_} fragment within {Mo_124_Ce_4_(histidine)_6_} depends strongly on the spatial arrangement of the coordinating histidine ligands and their hydrogen bonds formed with {Mo_8_}.^24^ The crystal structure analysis strongly indicates that all six histidine ligands must be protonated in order to minimize the repulsive electrostatic forces between the negatively charged assemblies (nanoring and {Mo_8_} cluster) and therefore increase system stability. Moreover, the orientation of the introduced {Mo_8_} fragment within {Mo_124_Ce_4_(histidine)_6_} depends strongly on the spatial arrangement of the coordinating histidine ligands and their hydrogen bonds formed with {Mo_8_}.^24^

The nonproteinogenic amino acid l-ornithine coordinates to {Mo_2_} units during the self-assembly processes and acts as a structure-directing ligand, triggering the formation of various guest@host architectures.^38^ Three cerium-containing nanoclusters were obtained using l-ornithine: the “Japanese rice-ball”-shaped α-{Mo_8_}@{Mo_124_Ce_4_(Orn)_6_}^38^ (Figure 6F), the elliptically shaped {Mo_17_}@{Mo_150_Ce_2_(Orn)_6_} (Figure 6G), and the elliptically β-{PMo_12_}@{Mo_150_Ce_2_(Orn)_6_}^38^ (Figure 6H). In all three nanowheels, six l-ornithine ligands are attached to the inner rim. In addition, the negatively charged polyoxomolybdate template cluster in the cavity of each nanowheel is stabilized by electrostatic forces and hydrogen bonds. {Mo_124_Ce_4_(Orn)_6_} encapsulates the in situ formed octamolybdate-type anion α-[Mo_8_O_26_]^4–^ (α-{Mo_8_}) (Figure 6F), whereas the nanoring {Mo_150_Ce_2_(Orn)_6_} can trap either the in situ formed [Mo_17_O_52_(H_2_O)_10_]^2–^ ({Mo_17_}) (Figure 6G) or the separately added Keggin-type anion β-[PMo_12_O_40_]^3–^ (β-{PMo_12_}) (Figure 6H).^38^ So far, {Mo_17_} has only been isolated as a template in {Mo_150_Ce_2_(Orn)_6_} (Figure 6G). In the scaffold {Mo_124_Ce_4_(Orn)_6_}, two Ce^III^ ions are located on each rim, separated by either one or two {Mo_2_} groups (Figure 6F). The scaffold {Mo_150_Ce_2_(Orn)_6_} contains a Ce^III^ ion on the upper and lower rim, located opposite to each other (Figure 6G).

## 4 Characterization of Ln-MBs in the Solid State

### 4.1 Single Crystal X-ray Diffraction

First and foremost, for new Ln-MB-based nanoclusters, it is essential to obtain suitable crystals for single crystal X-ray structure analysis to determine their three-dimensional shape and size in the solid state. The size, stability, scattering power, and quality of the crystal are critical for a single crystal X-ray structure analysis. Since Ln-MBs are composed of heavy atoms and thus produce a greater diffraction pattern than molecules containing just light atoms, the crystal to be examined can be significantly smaller than that of organic compounds. Crystals of Ln-MB-based nanoclusters are typically measured around 200 K and below rather than at room temperature. To generate Ln-MB crystals suitable for X-ray diffraction, the crystallization process may need to be slowed down (for example, by allowing the solvent to evaporate more slowly or by lowering the crystallization temperature), or the synthesis parameters have to be adjusted. The majority of Ln-MB synthesis optimizations are based on the “trial and error” concept, with the aim of finding the optimal starting component ratio.^32,37^

### 4.2 FTIR and Raman Spectroscopy, Powder Diffractometry, and TGA

**Figure 12:**
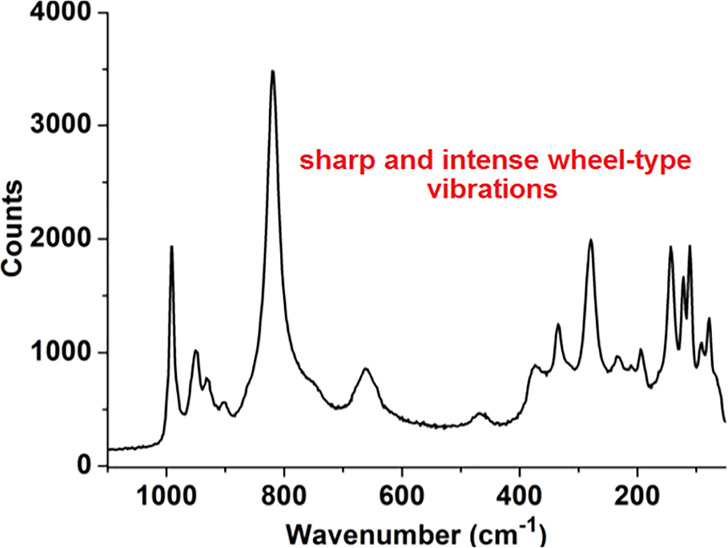
Raman spectrum of {Mo_100_Ce_6_}. Reprinted from ref (42). Copyright 2014 American Chemical Society.

Infrared spectroscopy displays characteristic bands, which appear <1100 cm^–1^ (Mo=O vibrations are located at higher wave numbers than Mo–O–Mo) and confirms only the presence of a polyoxometalate scaffold, whereas Raman spectroscopy shows characteristic bands for wheel-type scaffolds.^49^ Sharp bands are typically observed in the Raman spectra of MB/Ln-MBs at both low and high wavenumbers, which can be attributed to vibrations of {(Mo^V/VI^)_m_O} (m = 2,3,4) fragments (Figure 12) with the band at around 800 cm^–1^ being a symmetric stretching vibration of the equatorial {(Mo^V/VI^)_3_μ_3_-O} fragment.^19,50^ Raman spectroscopy also allows investigations of MBs/Ln-MBs in aqueous solution, since water molecules are only weakly Raman-active.

Structural characterization of MBs/LnMBs in the solid state is completed by powder diffractometry to evaluate their phase purity, and thermogravimetric analysis is conducted to assess their crystal water content (by heating the MB/Ln-MB containing sample typically up to ∼150 °C).

## 5 Stability and Speciation Studies of Ln-MBs in Solution

The stability and speciation of Ln-MBs must first be studied when dissolved before they can be used as possible molecules for applications in solution. How can the integrity of Ln-MBs be confirmed? How are aggregates, such as the spontaneous self-assembly of homogeneous, hollow spherical “blackberry” structures,^30^ identified? Which spectroscopic methods are relevant for the investigation of chirality in Ln-MBs? Is it feasible to show that the organic–inorganic hybridization in solution is preserved? Is it possible to determine the charge of these Ln-MB wheels at different pH values?

Among all the various investigation techniques for stability and speciation studies in solution, such as Raman-spectroscopy, ESI-MS and ES-IMS-MS, or UV–vis-Nir, small-angle X-ray scattering (SAXS) stands out when it comes to exploring nanoscopic structures (usually 1–100 nm) in solution.^51^ The utilization of SAXS, a highly powerful, nondestructive technique enables the determination of size, shape, and interactions of nanoscopic POMs and additionally allows for a variety of in situ analyses. Investigations of solution-phase speciation under various conditions (pH, conc., type of counterions, aged solutions, etc.) can be conducted in order to obtain information about their morphology and assembly when dissolved (e.g., is the POM oligomerized or present as a monomer? Is an ecliptically stacked monomeric arrangement or rather a shifted face-to-face dimeric configuration preferred?).^52,53^ Stability measurements can be performed to determine whether POMs are still intact under certain conditions (e.g., in catalytic processes).^51^ Fundamental understanding of reaction mechanisms^51^ can be provided and possible preferences for particular organic compounds and whether they accelerate the cluster formation of Ln-MBs can be examined.^47^ Furthermore, the feasibility of registering the start and maximum of nanosized cluster formation by using SAXS, enables the determination of critical reaction parameters for their synthesis.^54^ SAXS studies on Ln-MB systems have not yet been conducted.

### 5.1 ES-IMS-MS, Redox Titration, and UV–Vis-Nir

**Figure 13:**
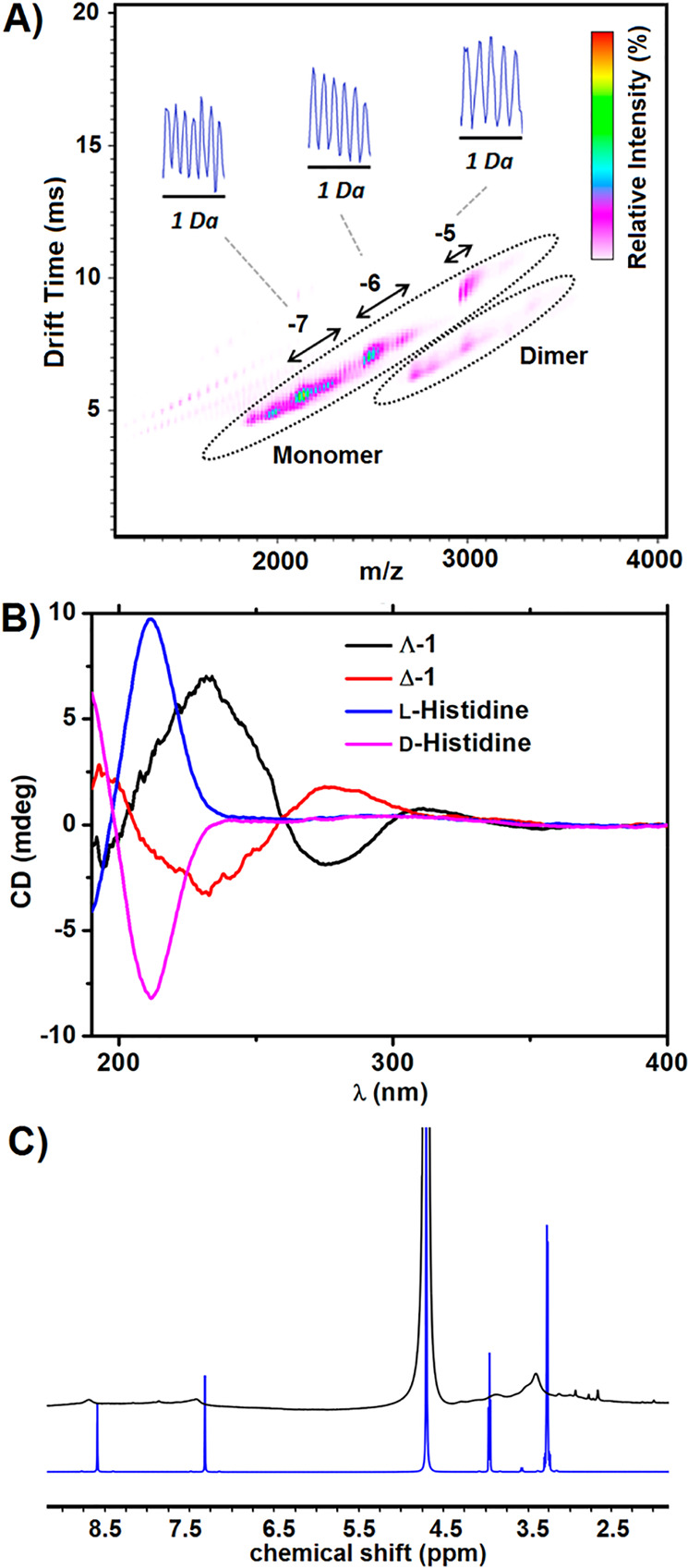
(A) ESI-IMS-MS of a solution containing the dimer {Mo_100_Ce_6_}_2_ with the dominating monomer {Mo_100_Ce_6_}.^42^ [Reprinted with from ref (42). Copyright 2014 American Chemical Society.] (B) CD spectra of Δ-{Mo_8_@Mo_124_Ce_4_(histidine)_6_}, Λ-{Mo_8_@Mo_124_Ce_4_(histidine)_6_}, l-histidine, and d-histidine.^24^ [Reprinted from ref (24). Copyright 2019, American Chemical Society.] C) ^1^H NMR spectra of {Mo_8_@Mo_124_Ce_4_(histidine)_6_} (black) and l-histidine (blue).^24^ [Reprinted from ref (24). Copyright 2019, American Chemical Society.]

Electrospray ionization-ion mobility spectrometry-mass spectrometry (ESI-IMS-MS) is an excellent method for investigating the speciation of gigantic MBs in solution/gas phase. ESI-IMS-MS is superior to ESI-MS for the following reason: MB/Ln-MB building blocks generate multiple species with fairly similar mass-to-charge ratios (*m*/*z*) in very different sizes/structures, resulting in overlapping envelopes in the mass spectra.^55^Figure 13A shows an ESI-IMS-MS spectrum of the dimer {Mo_100_Ce_6_}_2_ and the dominating monomer {Mo_100_Ce_6_}.^42^ The spectrum of the monomer {Mo_100_Ce_6_} was very similar to that of {Mo_100_Ce_6_}_2_ with the significant difference that no peak for the dimer was observed.

Redox titration is used to determine the number of Mo^V^ centers in the cluster (e.g., with a Ce^IV^ titration).^25^ However, sufficient solubility of the compound is required for a redox titration; otherwise, the Mo^V^ centers must be identified using bond valence sum calculations based on the crystal structure.

Solution stability of MB/Ln-MB-based nanoclusters can be monitored with UV–vis-Nir, as they show characteristic absorption bands from 600 to 1100 nm, which are attributed to the intervalence charge transfer between Mo^VI^ and Mo^V^.^17^

### 5.2 Circular Dichroism and ^1^H NMR Spectroscopy

Circular dichroism (CD) is suitable to determine if organic hybridization has occurred on chiral Ln-MBs and whether these are stable in solution. CD spectroscopy of Λ-{Mo_8_}@{Mo_124_Ce_4_(histidine)_6_} and Δ-{Mo_8_}@{Mo_124_Ce_4_(histidine)_6_} resulted in mutually mirror-inverted curves (Figure 13B) with a distinctive exciton splitting at 234 and 274 nm originating from histidine.^24^ A red shift of about 20 nm relative to unattached histidine strongly suggests coordination on the {Mo_124_Ce_4_} scaffold and, as a result, a restriction of the histidine’s free rotation.

^1^H NMR spectroscopy is well suited for examining hybridized inorganic–organic Ln-MBs for organic attachment, provided the molecule to be examined is sufficiently soluble in deuterated solvents. The ^1^H NMR analysis of {Mo_8_@Mo_124_Ce_4_(histidine)_6_} (Figure 13C) displays the characteristic signals for histidine, with the difference that the signals obtained are both broader and upfield- and downfield-shifted when compared to unattached histidine.^24^ The broadening of the signals as well as their upfield and downfield shift are probably caused by the shielding effect from the 24-electron reduced {Mo_124_Ce_4_}.^56^

### 5.3 “Blackberry”, Encapsulation Experiments, SLS, and DLS

Charged MB/Ln-MBs exhibit a unique behavior in solution, assembling into hollow, spherical blackberry-like aggregates.^30,31^ The interactions involved for their formation are counterion-mediated electrostatic attractions, van der Waals forces, and hydrogen bonding.^30^ A critical variable for the assembly of “blackberries” is the pH, which determines their size. Typically, the lower the pH, the smaller the overall negative charge (= weaker repulsive electrostatic interactions between adjacent anion clusters) and the larger the size of the “blackberry”.^30^ Encapsulation tests using the positively charged cetrimonium bromide (CTAB) surfactant allow for the investigation of the total Ln-MB charge. For {Mo_90_Ce_10_}, the charge could be determined at various pH levels (pH 0.5–2), indicating that {Mo_90_Ce_10_} exists in solution as a decaanionic cluster above pH 2.^31^ The concentration of the cations has the opposite effect, as demonstrated clearly by the increasing addition of divalent Ba^II^ cations, which exhibits enhanced attraction of macroion and counterion. The higher the concentration of Ba^II^ ions is, the larger the “blackberry” becomes, confirming that its assembly is a charge-regulated process.^31^ The first “blackberry” assembly of an Ln-MB was confirmed for {Mo_90_Ce_10_} (at pH 1 in the presence of Ba^II^) and verified by static light scattering (SLS).^31^ The hydrodynamic radius of {Mo_90_Ce_10_} was identified by the use of dynamic light scattering (DLS).^31^ POM superstructures, as well as structural changes induced by pH, counterion concentration, etc., can be observed and studied using SLS and DLS. Both light scattering techniques are able to determine dynamic properties of small POMs,^57^ such as the Keggin archetype, and gigantic assemblies.

## 6 The Influence of Lanthanides on the Redox Characteristics of Ln-MB Nanoclusters

The partial reduction of Mo^VI^-solutions results in Mo-scaffolds with a different degree of reduction (given in % in Table 3), which is calculated by dividing the number of Mo^V^ centers by the total number of Mo centers in the wheel (Table 3). When lanthanides are incorporated into MB scaffolds, the resulting Ln-MBs demonstrate almost the same degree of reduction as Ln-free MBs (Table 3) with Ln-MBs containing a slightly smaller number of Mo^V^ centers within their scaffold compared with Ln-free MBs, as they are typically made up of a smaller number of building blocks.^1,15,40^

**Table 3:** All MB/Ln-MB-Based Nanoclusters with Approximate Degree of Reduction (Number (*n*) of Accepted e^–^ Reflects the Number of Mo^V^ Centers in the Entire Wheel) Determined through Redox Titration^a^

MB/Ln-MB scaffold	*n* of accepted e^–^	% of reduction	examples of reported applications	ref
{Mo_36_}⊂{Mo_150_}	20	0 in {Mo_36_} and 13 in {Mo_150_}		(21)
{Mo_138_}	24	17		(20)
{Mo_154_}	28	18	cathode material	(19, 61)
{Mo_176_}	32	18		(1)
{Mo_180_(ornithine)_7_}	32	18		(35)
{Mo_150_}	28	19		(21)
{Mo_138_(acetate)_6_}	28	20	sensor technology	(20, 61)
{Mo_36_}_2_⊂{Mo_176_}	80	67 in {Mo_36_}_2_ and 18 in {Mo_176_}		(22)
{Mo_128_Eu_4_}	24	18		(44)
[Mo_130_Ce_6_}	24	18		(35)
{Mo_150_(La)_2_}	28	18		(31)
{Mo_96_La_8_}	20	19		(32)
{Mo_100_Ce_6_}	20	19		(33)
{Mo_120_Pr_6_}	24	19		(43)
{Mo_120_Ce_6_}	24	19		(41)
{Mo_120_La_6_}	24	19		(31)
{Mo_134_La_10_}	28	19		(34)
{Mo_90_Ce_10_}	20	20		(30)

a The structures are arranged in increasing degree of reduction of the MBs/LnMBs. The first half of the table contains MBs, and the second half contains Ln-MBs. The symbol “⊂” represents the inclusion of a template cluster within the wheel.

Mo^V^ centers, reflecting the scaffold’s degree of reduction, are integrated in Ln-MBs as Mo^V^–O–Mo^VI^ bridges, exactly as in MBs, and present in a considerable number within the Ln-MB scaffold. These Mo^V^–O–Mo^VI^ structural motifs are considered to be the catalytic centers, as proposed mechanistically in the {Mo_154_}-catalyzed oxidation of cyclohexane to cyclohexanol by Conte et al.^58^

A correlation has been established between the degree of reduction of gigantic Ln-free POMs and the yield of products in photoredox mediated organic coupling reactions. In the synthesis of 1,4-diphenyl substituted butane-1,4-dione, a yield of 87% was achieved with the keplerate {Mo_132_} (degree of reduction 45%) and a yield of 78% with the MB {Mo_154_} (degree of reduction 18%) as a catalyst.^59^ As Ln-MBs and MBs exhibit a comparable degree of reduction (Table 3), similar redox characteristics are to be expected.

## 7 Possible Applications and Relevance of Ln-MBs

### 7.1 Homogeneous and Heterogeneous Catalysis

The development of applications in materials using giant POMs, e.g., keplerates and [Mo_154–*x*_} is described in the recently published book chapter by Long and Cronin.^61^ While several intriguing examples of MBs as homogeneous and heterogeneous catalysts have been reported,^58,62−65^ merely one report about a Ln-MB showing catalytical behavior exists.^24^ However, based on their structure and stability in solution, potential applications can be foreseen. In homogeneous catalysis organosoluble POMs bearing lanthanides (e.g., the Wells–Dawson archetype) can exhibit chemoselectivity in organic reactions, such as in aldol, Diels–Alder-, Mannich-, and Mukaiyama-type reactions.^27,28^ Chiral hybridized complete Ln-MB ring systems can enclose nanosized guest molecules within their cavity,^24^ and as they contain lanthanides, which are Lewis acidic cations, these Ln^III^ ions might therefore enable the performance of (selective) catalytic reactions on entrapped substrates in their “Japanese rice-ball”-, circle-, egg-, and ellipse-shaped (see Figure 8) nanocavity and function as nanovessels for homogeneous catalysis in organic media. For their use in organic homogeneous catalysis, the Ln-MBs must be readily soluble in organic media. To increase the hydrophobicity of Ln-MBs, appropriate surface reagents can be used, which has already been implemented for the circular-shaped, lanthanide-free MB {Mo_176_}.^66^ Using the amphiphilic complexing agents didodecyldimethylammonium (DDMA) bromide and dioctadodecyldimethylammonium (DOMA) bromide, the hydrophobic aggregates Ln-MB-DDMA/DOMA could be obtained in which the rigid, hydrophilic, inorganic nanocluster would have been encapsulated by a flexible hydrophobic outer shell.

**Figure 14:**
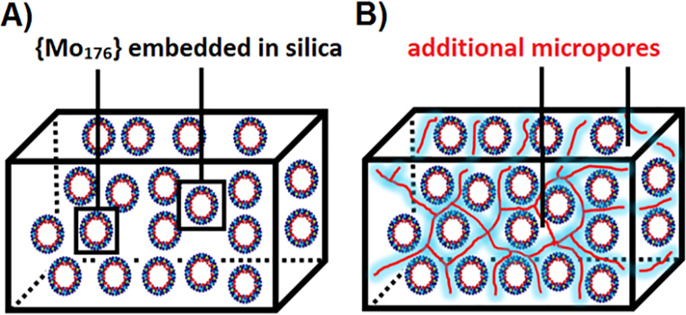
Schematic representation of Ln-MB-silica. (A) Embedding of {Mo_176_}^1^ in silica yields the hybrid material {Mo_176_}-silica.^67^ (B) {Mo_176_}-silica hybrid material with additional micropores created by PEG.^67^

In heterogeneous catalysis, MB nanorings have been shown to be effective oxidation catalysts^58,65^ and that they can be used as a solid solution.^67^ The chirality of Ln-MBs, caused with the help of Ln^III^ ions,^24^ could be exploited in heterogeneous catalysis, for instance, in asymmetric oxidation reactions. A Ln-MB-based solid solution for various oxidation reactions could be obtained as follows: (1) Ln-MBs could be embedded in silica using a sol–gel process as it has already been realized for the circular-shaped lanthanide-free MB {Mo_176_} (Figure 14).^67^ (2) Amorphous, optically clear objects (monoliths) of different sizes and shapes could be generated, creating a porous system that contains nanostructures with access to their inner surface (Figure 14). The synthesized Ln-MB-silica could behave like a solid solution because the incorporated POMs would be homogeneously distributed in the silica matrix. Additional micropores of different sizes could be created by adding polyethylene glycol (PEG) without causing a macroscopic phase separation or the release/decomposition of the POMs. As the pores introduced by PEG would be much smaller than the cavities of the POMs, molecules could diffuse into the POM, without the POM being released. These POM hybrids could be described as precisely defined, monodisperse reaction systems in which the amount of molybdenum remains constant regardless of the chemical process.

### 7.2 Molecular Recognition

**Figure 15:**
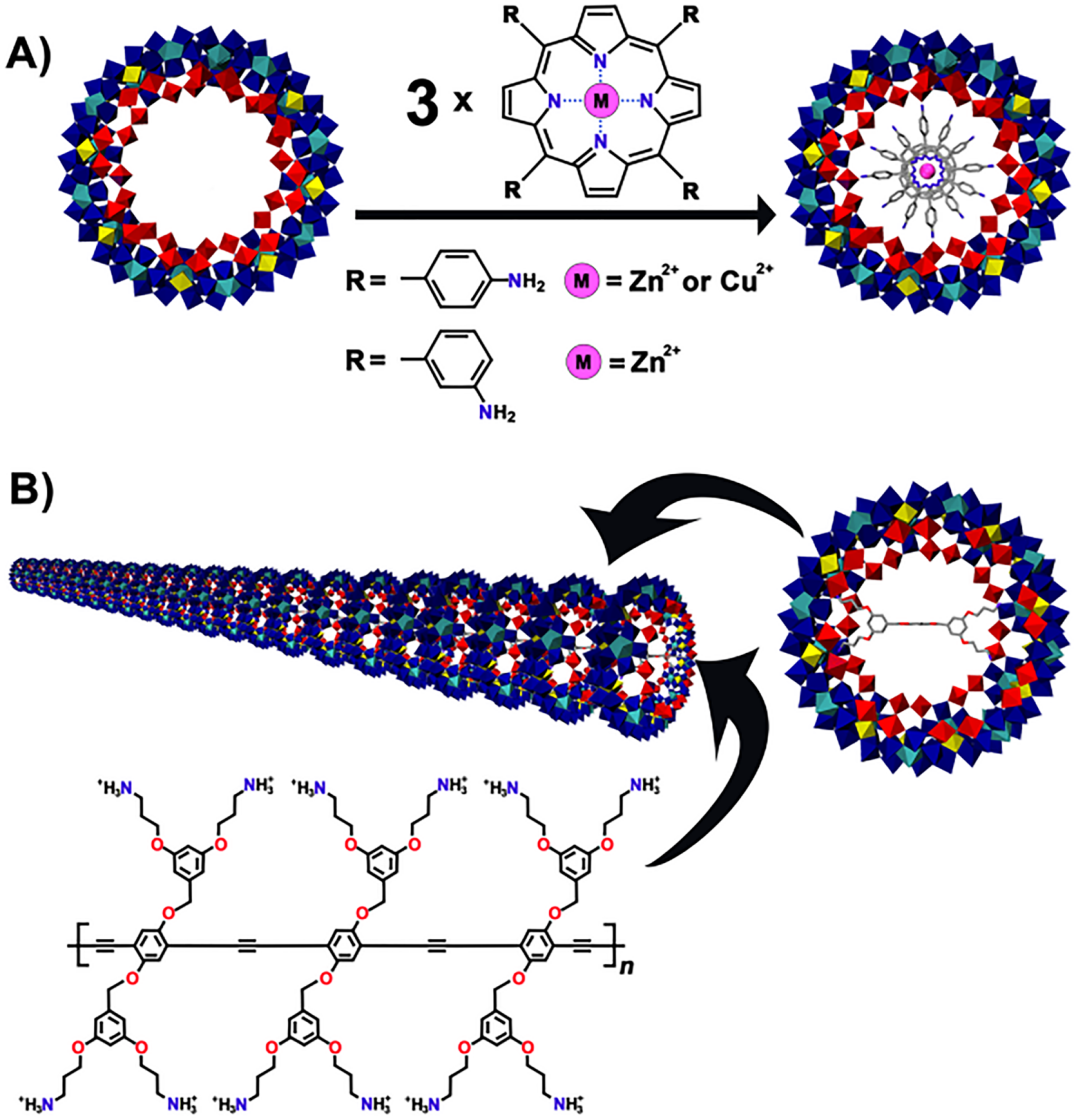
Schematic representation of {Mo_176_} complexating organic guest molecules selectively. (A) Inclusion of three metalloporphyrins with meso-aminophenyl substituents into the cavity of {Mo_176_}.^69^ Only the amino groups at the para- and meta-position of the phenyl ring form hydrogen bonds inside the wheel. (B) {Mo_176_} and the polymer *p*-phenylenebutadiynylene (PB_n_) forming an inorganic/organic polypseudorotaxane.^71^

Molecular recognitions are among the most significant processes of biological and supramolecular systems.^68^ Chiral Ln-MBs are supramolecular wheels with unique functional sites inside a well-defined cavity and, therefore, should be capable of chiral recognition and sensing in host–guest systems. The following findings indicate that Ln-MBs can be used for chiral recognition: (1) Chiral lanthanide-containing POMs are capable of conducting chiral recognition. This has been observed for the chiral POM [Ce(α_1_-P_2_W_17_O_61_)(H_2_O)_*x*_]^7–^ by chiral amino acids; the resulting stereoselective interactions were confirmed by ^13^P NMR spectroscopy.^29^ (2) Even achiral MBs can incorporate appropriate nanosized guests chemo-, regio-, and size-selectively^69^ (Figure 15A), and selective recognition will most likely be enhanced by introducing chirality into the system. (3) The interaction of biphenyl dicarboxylic acid (bridging ligand), cyclodextrin (chiral species), and the lanthanide Tb^III^ yielded polyrotaxane-type supramolecular assemblies, which enabled the recognition of small chiral molecules.^70^ Wheels can also form polyrotaxane-type supramolecular assemblies.^71^ The coassembly of the polymer *p*-phenylenebutadiynylene (PB_n_) (Figure 15B) with a MB yielded a one-dimensional, tubular arrangement of cofacially connected wheels. Both the rigidity and the pendant ammonium groups of the PB_n_ have a high affinity for the wheel surface, which lead to threading interactions with the wheel and the formation of so-called inorganic–organic polypseudorotaxanes. The threading interaction is so strong that even electrostatically fixed metalloporphyrins can be pushed out of the wheel.

Chiral Ln-MBs could also potentially serve as inorganic counterparts of cucurbit[*n*]urils, which are organic macrocyclic molecules composed of several glycoluril units and known for their outstanding recognition properties.^72,73^ In particular, the possibility to produce chiral Ln-MBs specially functionalized with different amino acids and peptide sequences makes them promising candidates for recognizing specific peptides and proteins. Thus, stable, highly scalable, and potentially more reliable synthetic receptors of inorganic–organic nature could be produced with the ability to specifically inhibit or enhance the biological activity of certain peptides or proteins. For example, smaller POM-peptide hybrids have been shown to inhibit the aggregation of β-amyloid peptides.^74^

### 7.3 Lanthanide Separation

**Figure 16:**
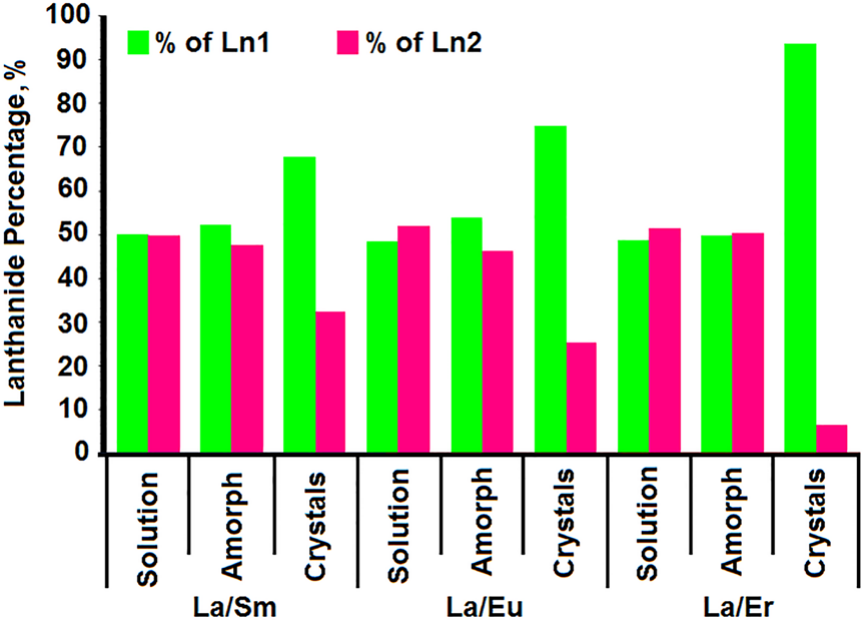
Comparison of the percentages of lanthanides in samples in various states obtained from binary lanthanide mixture in a thermodynamically controlled Ln-MB synthesis.^31^

Lanthanides are important metals in the design of special materials such as illuminant, colorants, permanent magnets, and lasers.^75−77^ They coexist quite often isostructural in sources of occurrence due to their chemical similarities^78−80^ (e.g., ion radii close together, lack of redox activity, etc.). As a result, the separation of neighboring 4f-elements in particular poses a significant challenge. A selective lanthanide separation based on purely inorganic chemical recognition has been realized through the crystallization of borates.^81^ The separation was based on the binding differences of the Ln^III^ ions. Remarkably, the thermodynamically controlled synthesis of the circular-shaped Ln-MBs {Mo_90_Ln_10_}^31^ (Ln^III^ = La^III^, Ce^III^, or Pr^III^) and {Mo_92_Ln_9_}^31^ (Ln^III^ = Nd^III^ or Sm^III^) (both depicted in Figure 5J and I) can be used to selectively enrich lanthanides from binary lanthanide mixtures as well.^31^ This was confirmed by ICP-OES analyses of lanthanide containing solid products formed in a reaction solution which contained two lanthanide species (Figure 16 shows the binary system La/Sm, Eu, or Er). The separation factor of various binary lanthanide mixtures is associated with the difference in ionic radius between the lanthanides; that is, the larger the difference in ionic radius, the better the separation. Therefore, lanthanides with large ionic radii are preferred for thermodynamically regulated wheel construction. Lanthanide separation is typically performed in industry using hydrometallurgical techniques; however, on a laboratory scale, the Ln-MB-based lanthanide separation method could prove to be an elegant approach.

## 8 Conclusion

The spectrum of Ln-MB wheel clusters is highly diverse, as they come in a variety of structural types, shapes (“Japanese rice-ball”, “egg”, “ellipse”, and “circle”) and sizes with an inner and outer diameter of 10–22 and 26–39 Å, respectively. These unique inorganic ring systems possess a nanometer-sized cavity that allows for unprecedented and intriguing host–guest chemistry. Specific scaffolds with the tuning of the nanometer-sized cavity can be conveniently achieved as all architecturally labile {Mo_2_}-type building blocks in Ln-MBs are both completely replaceable by lanthanide ions and organically modifiable. Not only the nanocavity but also the properties of the outer surface can be altered, specifically, by introducing organic surfactants to increase the hydrophobicity, which allows the adjustment of the cluster’s polarity. MBs with integrated lanthanide ions might be critical in applications such as catalysis, molecular recognition, and lanthanide ion separation, and as several spectroscopic techniques, including the powerful ESI-IMS-MS, have demonstrated, Ln-MBs preserve their integrity in solution, implying that they could endure robust reactions without decomposing. The archive of crystal structures of wheel-shaped Ln-MBs is still considerably expandable as many organic ligands, especially amine-containing ligands, remain to be explored. The conduction of SAXS studies of Ln-MBs in the solution phase is strongly recommended to explore their stability and speciation behavior in reactions and various media. We are confident that Ln-MBs will be used as an inorganic platform for the development of innovative molecular architectures and nanostructured materials as well as for the design of adaptive inorganic–organic chemical systems.

## References

[ref1] Müller A.; Krickemeyer E.; Bögge H.; Schmidtmann M.; Beugholt C.; Kögerler P.; Lu C. Formation of a Ring-Shaped Reduced “Metal Oxide” with the Simple Composition [(MoO_3_)_176_(H_2_O)_80_H_32_]. Angew. Chem., Int. Ed. 1998, 37, 1220–1223. 10.1002/(SICI)1521-3773(19980518)37:9<1220::AID-ANIE1220>3.0.CO;2-G. 29711236

[ref2] Müller A.; Gouzerh P. From linking of metal-oxide building blocks in a dynamic library to giant clusters with unique properties and towards adaptive chemistry. Chem. Soc. Rev. 2012, 41, 7431–7463. 10.1039/c2cs35169b. 22948798

[ref3] Shishido S.; Ozeki T. The pH dependent nuclearity variation of {Mo_154-*x*_}-type polyoxomolybdates and tectonic effect on their aggregations. J. Am. Chem. Soc. 2008, 130, 10588–10595. 10.1021/ja800784a. 18642905

[ref4] Gumerova N. I.; Rompel A. Polyoxometalates in solution: Speciation under spotlight. Chem. Soc. Rev. 2020, 49, 7568–7601. 10.1039/D0CS00392A. 32990698

[ref5] Cruywagen J. J.; Draaijer A. G.; Heyns J. B. B.; Rohwer E. A. Molybdenum (VI) equilibria in different ionic media. Formation constants and thermodynamic quantities. Inorg. Chim. Acta 2002, 331, 322–329. 10.1016/S0020-1693(02)00700-4.

[ref6] Howarth O. W.; Kelly P.; Pettersson L. Aqueous isopolymolybdates(VI): an oxygen-17 and molybdenum-95 nuclear magnetic resonance study. J. Chem. Soc., Dalton Trans. 1990, 81–84. 10.1039/dt9900000081.

[ref7] Maksimovskaya R. I.; Maksimov G. M. ^95^Mo and ^17^O NMR studies of aqueous molybdate solutions. Inorg. Chem. 2007, 46, 3688–3695. 10.1021/ic061549n. 17385850

[ref8] Tytko K.-H.; Glemser O. Isopolymolybdates and isopolytungstates. Adv. Inorg. Chem. Radiochem. 1976, 19, 239–315. 10.1016/S0065-2792(08)60073-4.

[ref9] Alyea E. C.; Topich J. Molybdenum-95 NMR spectra of dioxomolybdenum(VI) schiff base complexes. Inorg. Chim. Acta 1982, 65, L95–L96. 10.1016/S0020-1693(00)93505-9.

[ref10] Christensen K. A.; Miller P. E.; Minelli M.; Rockway T.W.; Enemark J. H. Mo NMR spectra of Dioxomolybdenum (VI) complexes. Inorg. Chim. Acta 1981, 56, L27–L28. 10.1016/S0020-1693(00)88521-7.

[ref11] Cruywagen J. J.; Heyns J. B. B. Molybdenum(VI) equilibria at high perchloric acid concentration. Polyhedron 2000, 19, 907–911. 10.1016/S0277-5387(00)00331-4.

[ref12] Kierkegaard P. Oxide compounds of P and Me or W. Ark. Kemi 1962, 19, 51–74.

[ref13] Minelli M.; Yamanouchi K.; Enemark J. H.; Subramanian P.; Kaul B. B.; Spence J. T. Molybdenum-95 NMR measurements of dioxomolybdenum(VI) complexes. 3. Inverse halogen dependence of molybdenum chemical shift of [MoO_2_]^2+^ complexes. Inorg. Chem. 1984, 23, 2554–2556. 10.1021/ic00184a037.

[ref14] Schulz I. Über einige neue Phosphorsäureverbindungen des 6-wertigen Wolframs und Molybdäns. Z. Anorg. Allg. Chem. 1955, 281, 99–112. 10.1002/zaac.19552810110.

[ref15] Paulat-Böschen I. X-Ray crystallographic determination of the structure of the isopolyanion [Mo_36_O_112_(H_2_O)_16_]^8–^ in the compound K_8_[Mo_36_O_112_ (H_2_O)_16_]·36H_2_O. J. Chem. Soc., Chem. Commun. 1979, 780–782. 10.1039/C39790000780.

[ref16] Müller A.; Roy S. Metal-oxide based nanoobjects: reactivity, building blocks for polymeric structures and structural variety. Russ. Chem. Rev. 2002, 71, 981–991. 10.1070/RC2002v071n12ABEH000751.

[ref17] Müller A.; Serain C. Soluble molybdenum blues “des Pudels Kern”. Acc. Chem. Res. 2000, 33, 2–10. 10.1021/ar9601510. 10639070

[ref18] Scheele C. W. Sämtliche Physische und Chemische Werke; Hermbstädt D. S. F., Ed.; Martin Sänding OHG: Niederwalluf/Wiesbaden, 1793; Vol. 1, pp 185–200 (reprint: original 1793).

[ref19] Müller A.; Meyer J.; Krickemeyer E.; Diemann E. Molybdenum blue: a 200 year old mystery unveiled. Angew. Chem., Int. Ed. 1996, 35, 1206–1208. 10.1002/anie.199612061.

[ref20] Müller A.; Maiti R.; Schmidtmann M.; Bögge H.; Das S. K.; Zhang W. Mimicking oxide surfaces: different types of defects and ligand coordination at well defined positions of a molybdenum oxide based nanocluster. Chem. Commun. 2001, 2126–2127. 10.1039/b106092a. 12240195

[ref21] Miras H. N.; Richmond C. J.; Long D.-L.; Cronin L. Solution-phase monitoring of the structural evolution of a molybdenum blue nanoring. J. Am. Chem. Soc. 2012, 134, 3816–3824. 10.1021/ja210206z. 22257105

[ref22] Müller A.; Shah S. Q.; Bögge H.; Schmidtmann M. Molecular growth from a Mo_176_ to a Mo_248_ cluster. Nature 1999, 397, 48–50. 10.1038/16215.

[ref23] Müller A.; Das S. K.; Kuhlmann C.; Bögge H.; Schmidtmann M.; Diemann E.; Krickemeyer E.; Hormes J.; Modrow H.; Schindler M. On the option of generating novel type surfaces with multiphilic ligands within the cavity of a giant metal–oxide based wheel type cluster: chemical reactions with well-defined nanoobjects. Chem. Commun. 2001, 655–656. 10.1039/b100362n.

[ref24] Xuan W.; Pow R.; Watfa N.; Zheng Q.; Surman A. J.; Long D.-L.; Cronin L. Stereoselective Assembly of Gigantic Chiral Molybdenum Blue Wheels Using Lanthanide Ions and Amino Acids. J. Am. Chem. Soc. 2019, 141, 1242–1250. 10.1021/jacs.8b09750. 30495944 PMC6351008

[ref25] Müller A.; Beugholt C.; Bögge H.; Schmidtmann M. Influencing the Size of Giant Rings by Manipulating Their Curvatures: Na_6_[Mo_120_O_366_(H_2_O)_48_H_12_{Pr(H_2_O)_5_}_6_]·(∼200H_2_O) with Open Shell Metal Centers at the Cluster Surface. Inorg. Chem. 2000, 39, 3112–3113. 10.1021/ic000168l. 11196845

[ref26] Barrett A. G. M.; Braddock D. C. Scandium(III) or lanthanide(III) triflates as recyclable catalysts for the direct acetylation of alcohols with acetic acid. Chem. Commun. 1997, 351–352. 10.1039/a606484a.

[ref27] Boglio C.; Lemière G.; Hasenknopf B.; Thorimbert S.; Lacôte E.; Malacria M. Lanthanide complexes of the monovacant Dawson polyoxotungstate [α_1_-P_2_W_17_O_61_]^10–^ as selective and recoverable lewis acid catalysts. Angew. Chem., Int. Ed. 2006, 45, 3324–3327. 10.1002/anie.200600364. 16619320

[ref28] Dupré N.; Rémy P.; Micoine K.; Boglio C.; Thorimbert S.; Lacôte E.; Hasenknopf B.; Malacria M. Chemoselective catalysis with organosoluble Lewis acidic polyoxotungstates. Chem.–Eur. J. 2010, 16, 7256–7264. 10.1002/chem.201000411. 20455223

[ref29] Sadakane M.; Dickman M. H.; Pope M. T. Chiral Polyoxotungstates. 1. Stereoselective Interaction of Amino Acids with Enantiomers of [Ce^III^(α_1_-P_2_W_17_O_61_)(H_2_O)_x_]^7-^. The Structure of DL-[Ce_2_(H_2_O)_8_ (P_2_W_17_O_61_)_2_]^14-^. Inorg. Chem. 2001, 40, 2715–2719. 10.1021/ic0014383. 11375685

[ref30] Liu T.; Diemann E.; Li H.; Dress A. W. M.; Müller A. Self-assembly in aqueous solution of wheel-shaped Mo_154_ oxide clusters into vesicles. Nature 2003, 426, 59–62. 10.1038/nature02036. 14603315

[ref31] Garrido Ribó E.; Bell N. L.; Xuan W.; Luo J.; Long D.-L.; Liu T.; Cronin L. Synthesis, Assembly, and Sizing of Neutral, Lanthanide Substituted Molybdenum Blue Wheels {Mo_90_Ln_10_}. J. Am. Chem. Soc. 2020, 142, 17508–17514. 10.1021/jacs.0c07146. 32965108

[ref32] Xuan W.; Pow R.; Long D. L.; Cronin L. Exploring the Molecular Growth of Two Gigantic Half-Closed Polyoxometalate Clusters {Mo_180_} and {Mo_130_Ce_6_}. Angew. Chem., Int. Ed. 2017, 56, 9727–9731. 10.1002/anie.201702957. PMC5600119 28508585

[ref33] Nakamura I.; Miras H. N.; Fujiwara A.; Fujibayashi M.; Song Y.-F.; Cronin L.; Tsunashima R. Investigating the formation of “Molybdenum Blues” with gel electrophoresis and mass spectrometry. J. Am. Chem. Soc. 2015, 137, 6524–6530. 10.1021/ja512758j. 25897816

[ref34] Müller A.; Roy S. En route from the mystery of molybdenum blue via related manipulatable building blocks to aspects of materials science. Coord. Chem. Rev. 2003, 245, 153–166. 10.1016/S0010-8545(03)00110-3.

[ref35] Baker L. C. W.; Glick D. C. Present General Status of Understanding of Heteropoly Electrolytes and a Tracing of Some Major Highlights in the History of Their Elucidation. Chem. Rev. 1998, 98, 3–50. 10.1021/cr960392l. 11851498

[ref36] Gumerova N. I.; Rompel A. Synthesis, structures and applications of electron-rich polyoxometalates. Nat. Rev. Chem. 2018, 2, 0112 10.1038/s41570-018-0112.

[ref37] Duros V.; Grizou J.; Xuan W.; Hosni Z.; Long D. L.; Miras H. N.; Cronin L. Human versus robots in the discovery and crystallization of gigantic polyoxometalates. Angew. Chem., Int. Ed. 2017, 56, 10815–10820. 10.1002/anie.201705721. PMC5577512 28649740

[ref38] Xuan W.; Pow R.; Zheng Q.; Watfa N.; Long D. L.; Cronin L. Ligand-Directed Template Assembly for the Construction of Gigantic Molybdenum Blue Wheels. Angew. Chem., Int. Ed. 2019, 58, 10867–10872. 10.1002/anie.201901818. PMC6771582 31155800

[ref39] She S.; Xuan W.; Bell N. L.; Pow R.; Ribo E. G.; Sinclair Z.; Long D.-L.; Cronin L. Peptide sequence mediated self-assembly of molybdenum blue nanowheel superstructures. Chem. Sci. 2021, 12, 2427–2432. 10.1039/D0SC06098D. PMC8179307 34164008

[ref40] Yamase T.; Ishikawa E.; Abe Y.; Yano Y. Photoinduced self-assembly to lanthanide-containing molybdenum-blue superclusters and molecular design. J. Alloys Compd. 2006, 408, 693–700. 10.1016/j.jallcom.2004.11.099.

[ref41] Yamase T.; Kumagai S.; Prokop P. V.; Ishikawa E.; Tomsa A.-R. {Mo_96_La_8_} Eggshell Ring and Self-Assembly to {Mo_132_} Keplerate through Mo-blue Intermediate, Involved in UV-Photolysis of [Mo_7_O_24_]^6–^/Carboxylic Acid System at pH 4. Inorg. Chem. 2010, 49, 9426–9437. 10.1021/ic101027d. 20853895

[ref42] Xuan W.; Surman A. J.; Miras H. N.; Long D.-L.; Cronin L. Controlling the ring curvature, solution assembly, and reactivity of gigantic molybdenum blue wheels. J. Am. Chem. Soc. 2014, 136, 14114–14120. 10.1021/ja5062483. 25188897

[ref43] Ishikawa E.; Yano Y.; Yamase T. Coordination of {Mo_142_} Ring to La^3+^ Provides Elliptical {Mo_134_La_10_} Ring with a Variety of Coordination Modes. Materials 2010, 3, 64–75. 10.3390/ma3010064.

[ref44] Cronin L.; Beugholt C.; Krickemeyer E.; Schmidtmann M.; Bögge H.; Kögerler P.; Luong T. K. K.; Müller A. “Molecular Symmetry Breakers” Generating Metal-Oxide-Based Nanoobject Fragments as Synthons for Complex Structures: [{Mo_128_Eu_4_O_388_H_10_(H_2_O)_81_}_2_]^20–^, a Giant-Cluster Dimer. Angew. Chem., Int. Ed. 2002, 41, 2805–2808. 10.1002/1521-3773(20020802)41:15<2805::AID-ANIE2805>3.0.CO;2-E. 12203492

[ref45] Yamase T.; Yano Y.; Ishikawa E. Photoreductive self-assembly from [Mo_7_O_24_]^6-^ to carboxylates-coordinated {Mo_142_} Mo-blue nanoring in the presence of carboxylic acids. Langmuir 2005, 21, 7823–7832. 10.1021/la051301v. 16089388

[ref46] Kubota M. Decomposition of oxalic acid with nitric acid. J. Radioanal. Nucl. Chem. 1982, 75, 39–49. 10.1007/BF02519972.

[ref47] Falaise C.; Khlifi S.; Bauduin P.; Schmid P.; Shepard W.; Ivanov A. A.; Sokolov M. N.; Shestopalov M. A.; Abramov P. A.; Cordier S.; et al. “Host in Host” Supramolecular Core–Shell Type Systems Based on Giant Ring-Shaped Polyoxometalates. Angew. Chem., Int. Ed. 2021, 60, 14146–14153. 10.1002/anie.202102507. 33724635

[ref48] Niven M. L.; Cruywagen J. J.; Heyns J. B. B. The first observation of γ-octamolybdate: synthesis, crystal and molecular structure of [Me_3_N(CH_2_)_6_NMe_3_]_2_[Mo_8_O_26_]·2H_2_O. J. Chem. Soc., Dalton Trans. 1991, 2007–2011. 10.1039/DT9910002007.

[ref49] Botar B.; Ellern A.; Kögerler P. Mapping the formation areas of giant molybdenum blue clusters: a spectroscopic study. Dalton Trans. 2012, 41, 8951–8959. 10.1039/c2dt30661a. 22717474

[ref50] Müller A.; Das S. K.; Fedin V. P.; Krickemeyer E.; Beugholt C.; Bögge H.; Schmidtmann M.; Hauptfleisch B. Rapid and Simple Isolation of the Crystalline Molybdenum-Blue Compounds with Discrete and Linked Nanosized Ring-Shaped Anions: Na_15_[MoMo O_462_H_14_(H_2_O)_70_]_0.5_[MoMoO_457_H_14_(H_2_O)_68_]_0.5_·ca.400H_2_O and Na_22_[MoMoO_442_H_14_(H_2_O)_58_]·ca.250H_2_O. Z. Anorg. Allg. Chem. 1999, 625, 1187–1192. 10.1002/(SICI)1521-3749(199907)625:7<1187::AID-ZAAC1187>3.0.CO;2-#.

[ref51] Nyman M. Small-angle X-ray scattering to determine solution speciation of metal-oxo clusters. Coord. Chem. Rev. 2017, 352, 461–472. 10.1016/j.ccr.2016.11.014.

[ref52] Hou Y.; Zakharov L. N.; Nyman M. Observing assembly of complex inorganic materials from polyoxometalate building blocks. J. Am. Chem. Soc. 2013, 135, 16651–16657. 10.1021/ja4086484. 24116690

[ref53] Colliard I.; Nyman M. Building [U^IV^_70_(OH)_36_ (O)_64_]^4–^ Oxocluster Frameworks with Sulfate, Transition Metals, and U^V^. Chem.–Eur. J. 2020, 26, 12481–12488. 10.1002/chem.202002403. 32609912

[ref54] Yin P.; Wu B.; Li T.; Bonnesen P. V.; Hong K.; Seifert S.; Porcar L.; Do C.; Keum J. K. Reduction-triggered self-assembly of nanoscale molybdenum oxide molecular clusters. J. Am. Chem. Soc. 2016, 138, 10623–10629. 10.1021/jacs.6b05882. 27459601

[ref55] Robbins P. J.; Surman A. J.; Thiel J.; Long D.-L.; Cronin L. Use of ion-mobility mass spectrometry (IMS-MS) to map polyoxometalate Keplerate clusters and their supramolecular assemblies. Chem. Commun. 2013, 49, 1909–1911. 10.1039/c3cc38615e. 23364185

[ref56] Zhang L.; Li Y.; Zhou Y. Surface modification with multiphilic ligands at detectable well defined active positions of nano-object of giant wheel shaped molybdenum blue showing third-order nonlinear optical properties. J. Mol. Struct. 2010, 969, 69–74. 10.1016/j.molstruc.2010.01.043.

[ref57] Malinenko A.; Jonchère A.; Girard L.; Parrès-Maynadié S.; Diat O.; Bauduin P. Are Keggin’s POMs charged nanocolloids or multicharged anions?. Langmuir 2018, 34, 2026–2038. 10.1021/acs.langmuir.7b03640. 29278508

[ref58] Conte M.; Liu X.; Murphy D. M.; Taylor S. H.; Whiston K.; Hutchings G. J. Insights into the reaction mechanism of cyclohexane oxidation catalysed by molybdenum blue nanorings. Catal. Lett. 2016, 146, 126–135. 10.1007/s10562-015-1660-y.

[ref59] Das S.; Lai D.; Mallick A.; Roy S. Photo Redox Mediated Inexpensive One-Pot Synthesis of 1, 4-Diphenyl Substituted Butane-1, 4-Dione from Styrene using Polyoxometalate as a Catalyst. Chemistry Select 2016, 1, 691–695. 10.1002/slct.201500052.

[ref61] Long D.-L.; Cronin L. Advances in gigantic polyoxomolybdate chemistry. Adv. Inorg. Chem. 2021, 78, 227–267. 10.1016/bs.adioch.2021.06.003.

[ref62] Chen X.; Zhang G.; Li B.; Wu L. An integrated giant polyoxometalate complex for photothermally enhanced catalytic oxidation. Sci. Adv. 2021, 7, eabf8413 10.1126/sciadv.abf8413. 34301598 PMC8302132

[ref64] Das S.; Biswas S.; Balaraju T.; Barman S.; Pochamoni R.; Roy S. Photochemical reduction of carbon dioxide coupled with water oxidation using various soft-oxometalate (SOM) based catalytic systems. J. Mater. Chem. A 2016, 4, 8875–8887. 10.1039/C6TA02825J.

[ref65] Liu X.; Conte M.; Weng W.; He Q.; Jenkins R. L.; Watanabe M.; Morgan D. J.; Knight D. W.; Murphy D. M.; Whiston K.; et al. Molybdenum blue nano-rings: an effective catalyst for the partial oxidation of cyclohexane. Catal. Sci. Technol. 2015, 5, 217–227. 10.1039/C4CY01213E.

[ref66] Polarz S.; Smarsly B.; Antonietti M. Colloidal Organization and Clusters: Self-Assembly of Polyoxometalate-Surfactant Complexes towards Three-Dimensional Organized Structures. ChemPhysChem 2001, 2, 457–461. 10.1002/1439-7641(20010716)2:7<457::AID-CPHC457>3.0.CO;2-#. 23696531

[ref67] Polarz S.; Smarsly B.; Göltner C.; Antonietti M. The Interplay of Colloidal Organization and Oxo-Cluster Chemistry: Polyoxometalate–Silica Hybrids—Materials with a Nanochemical Function. Adv. Mater. 2000, 12, 1503–1507. 10.1002/1521-4095(200010)12:20<1503::AID-ADMA1503>3.0.CO;2-X.

[ref68] Ariga K.; Ito H.; Hill J. P.; Tsukube H. Molecular recognition: from solution science to nano/materials technology. Chem. Soc. Rev. 2012, 41, 5800–5835. 10.1039/c2cs35162e. 22773130

[ref69] Tsuda A.; Hirahara E.; Kim Y.-S.; Tanaka H.; Kawai T.; Aida T. A molybdenum crown cluster forms discrete inorganic–organic nanocomposites with metalloporphyrins. Angew. Chem., Int. Ed. 2004, 43, 6327–6331. 10.1002/anie.200460990. 15389887

[ref70] Yoshihara D.; Tsuchiya Y.; Noguchi T.; Yamamoto T.; Dawn A.; Shinkai S. Cyclodextrin-Assisted Synthesis of a Metallosupramolecular Terbium(III) Polymer and Its Fluorescence Properties and Chiral Recognition. Chem.–Eur. J. 2013, 19, 15485–15488. 10.1002/chem.201302138. 24127417

[ref71] Alam M. A.; Kim Y.-S.; Ogawa S.; Tsuda A.; Ishii N.; Aida T. Directed 1D Assembly of a Ring-Shaped Inorganic Nanocluster Templated by an Organic Rigid-Rod Molecule: An Inorganic/Organic Polypseudorotaxane. Angew. Chem., Int. Ed. 2008, 47, 2070–2073. 10.1002/anie.200705308. 18257008

[ref72] Lagona J.; Mukhopadhyay P.; Chakrabarti S.; Isaacs L. The cucurbit[*n*]uril family. Angew. Chem., Int. Ed. 2005, 44, 4844–4870. 10.1002/anie.200460675. 16052668

[ref73] Smith L. C.; Leach D. G.; Blaylock B. E.; Ali O. A.; Urbach A. R. Sequence-specific, nanomolar peptide binding via cucurbit[8]uril-induced folding and inclusion of neighboring side chains. J. Am. Chem. Soc. 2015, 137, 3663–3669. 10.1021/jacs.5b00718. 25710854

[ref74] Li M.; Xu C.; Wu L.; Ren J.; Wang E.; Qu X. Self-Assembled Peptide–Polyoxometalate Hybrid Nanospheres: Two in One Enhances Targeted Inhibition of Amyloid β-Peptide Aggregation Associated with Alzheimer’s Disease. Small 2013, 9, 3455–3461. 10.1002/smll.201202612. 23650245

[ref75] Binnemans K. Lanthanide-based luminescent hybrid materials. Chem. Rev. 2009, 109, 4283–4374. 10.1021/cr8003983. 19650663

[ref76] Shibasaki M.; Yoshikawa N. Lanthanide complexes in multifunctional asymmetric catalysis. Chem. Rev. 2002, 102, 2187–2210. 10.1021/cr010297z. 12059266

[ref77] Woodruff D. N.; Winpenny R. E. P.; Layfield R. A. Lanthanide single-molecule magnets. Chem. Rev. 2013, 113, 5110–5148. 10.1021/cr400018q. 23550940

[ref78] Cotton S. Lanthanide and actinide chemistry; John Wiley & Sons: 2013.

[ref79] Löble M. W.; Keith J. M.; Altman A. B.; Stieber S. C. E.; Batista E. R.; Boland K. S.; Conradson S. D.; Clark D. L.; Lezama Pacheco J.; Kozimor S. A.; et al. Covalency in lanthanides. An X-ray absorption spectroscopy and density functional theory study of LnCl_6_^x–^ (x = 3, 2). J. Am. Chem. Soc. 2015, 137, 2506–2523. 10.1021/ja510067v. 25689484

[ref80] Minasian S. G.; Krinsky J. L.; Rinehart J. D.; Copping R.; Tyliszczak T.; Janousch M.; Shuh D. K.; Arnold J. A Comparison of 4 f vs 5 f Metal– Metal Bonds in (CpSiMe_3_)_3_M–ECp* (M = Nd, U; E= Al, Ga; Cp*= C_5_Me_5_): Synthesis, Thermodynamics, Magnetism, and Electronic Structure. J. Am. Chem. Soc. 2009, 131, 13767–13783. 10.1021/ja904565j. 19725526

[ref81] Yin X.; Wang Y.; Bai X.; Wang Y.; Chen L.; Xiao C.; Diwu J.; Du S.; Chai Z.; Albrecht-Schmitt T. E.; Wang S. Rare earth separations by selective borate crystallization. Nat. Commun. 2017, 8, 14438 10.1038/ncomms14438. 28290448 PMC5355876

